# Iron oxide and iron oxyhydroxide nanoparticles impair SARS-CoV-2 infection of cultured cells

**DOI:** 10.1186/s12951-022-01542-2

**Published:** 2022-07-30

**Authors:** Marta L. DeDiego, Yadileiny Portilla, Neus Daviu, Darío López-García, Laura Villamayor, Vladimir Mulens-Arias, Jesús G. Ovejero, Álvaro Gallo-Cordova, Sabino Veintemillas-Verdaguer, M. Puerto Morales, Domingo F. Barber

**Affiliations:** 1 Department of Molecular and Cellular Biology, Centro Nacional de Biotecnología (CNB-CSIC), Darwin 3, 28049 Madrid, Spain; 2 Department of Immunology, Oncology and Nanobiomedicine Initiative, Centro Nacional de Biotecnología (CNB-CSIC), Darwin 3, 28049 Madrid, Spain; 3 Department of Energy, Environment and Health, Instituto de Ciencia de Materiales de Madrid (ICMM-CSIC), Sor Juana Inés de la Cruz 3, 28049 Madrid, Spain; 4 Present Address: Instituto de Investigaciones Biomédicas “Alberto Sols” (IIBm-CSIC-UAM), Arturo Duperier 4, 28029 Madrid, Spain; 5 Present Address: Department of Experimental and Health Sciences (DCEXS), Integrative Biomedical Materials and Nanomedicine Lab, Pompeu Fabra University, PRBB, Carrer Doctor Aiguader 88, 08003 Barcelona, Spain; 6 Department of Dosimetry and Radioprotection, General University Hospital Gregorio Marañón, Dr Esquerdo 46, 28007 Madrid, Spain

**Keywords:** Iron oxide nanoparticles, Iron oxyhydroxide nanoparticles, SARS-CoV-2, Viral infection, Viral replication, Oxidative stress, Iron metabolism, Anti-anemic, MRI contrast agents

## Abstract

**Background:**

Coronaviruses usually cause mild respiratory disease in humans but as seen recently, some human coronaviruses can cause more severe diseases, such as the Severe Acute Respiratory Syndrome Coronavirus 2 (SARS-CoV-2), the global spread of which has resulted in the ongoing coronavirus pandemic.

**Results:**

In this study we analyzed the potential of using iron oxide nanoparticles (IONPs) coated with biocompatible molecules like dimercaptosuccinic acid (DMSA), 3-aminopropyl triethoxysilane (APS) or carboxydextran (FeraSpin™ R), as well as iron oxyhydroxide nanoparticles (IOHNPs) coated with sucrose (Venofer^®^), or iron salts (ferric ammonium citrate -FAC), to treat and/or prevent SARS-CoV-2 infection. At non-cytotoxic doses, IONPs and IOHNPs impaired virus replication and transcription, and the production of infectious viruses in vitro, either when the cells were treated prior to or after infection, although with different efficiencies. Moreover, our data suggest that SARS-CoV-2 infection affects the expression of genes involved in cellular iron metabolism. Furthermore, the treatment of cells with IONPs and IOHNPs affects oxidative stress and iron metabolism to different extents, likely influencing virus replication and production. Interestingly, some of the nanoparticles used in this work have already been approved for their use in humans as anti-anemic treatments, such as the IOHNP Venofer^®^, and as contrast agents for magnetic resonance imaging in small animals like mice, such as the FeraSpin™ R IONP.

**Conclusions:**

Therefore, our results suggest that IONPs and IOHNPs may be repurposed to be used as prophylactic or therapeutic treatments in order to combat SARS-CoV-2 infection.

**Graphical Abstract:**

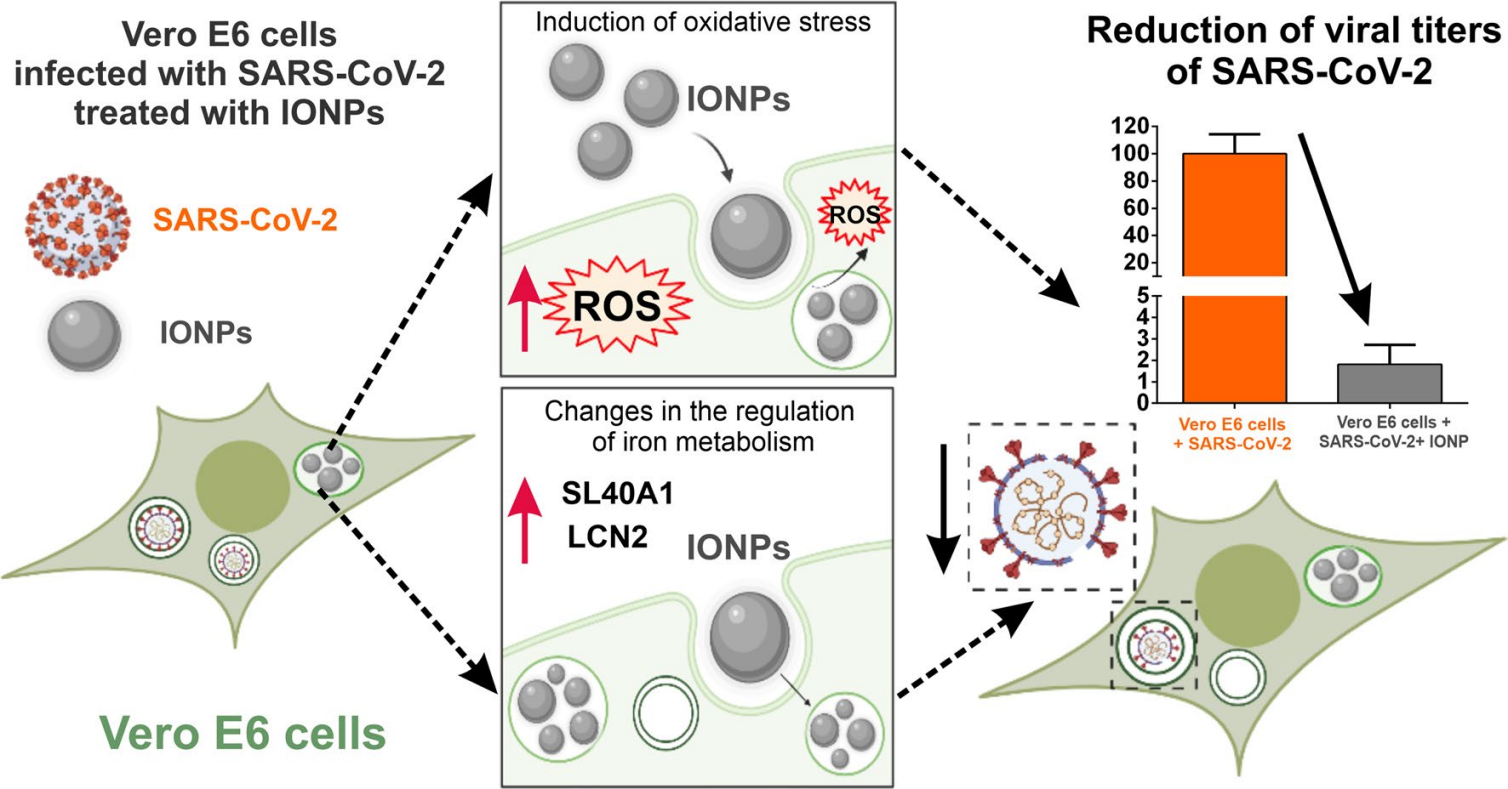

**Supplementary Information:**

The online version contains supplementary material available at 10.1186/s12951-022-01542-2.

## Background

Coronaviruses are viruses of the Coronaviridae family with a positive-sense, single-stranded RNA genome [1] and a lipid envelope, and they generally cause mild respiratory disease in humans [1, 2]. However, some members of the family can cause severe respiratory diseases in infected individuals, such as the Severe Acute Respiratory Syndrome Coronavirus 1 and 2 (SARS-CoV and SARS-CoV-2, respectively), and the Middle East Respiratory Syndrome Coronavirus (MERS-CoV), zoonotic viruses that emerged in humans from animals (most likely bats) [1, 3, 4]. In December 2019, a new infectious coronavirus disease (COVID-19) was first identified in Wuhan, the capital of China’s Hubei province, caused by the SARS-CoV-2 virus (genetically similar to SARS-CoV). This disease has since spread globally to provoke the ongoing coronavirus pandemic [5], which as of March 2022 has caused over 6 million deaths worldwide, with at least 448 million people infected [6]. While silent or provoking only mild symptoms in the majority of cases, such as fever, cough, sore throat, loss of taste or smell, muscle pain and diarrhea, SARS-CoV-2 may lead to a more severe COVID-19 that is primarily but not exclusively characterized by severe pneumonia or acute respiratory distress syndrome (ARDS) [7]. Other complications associated with the cardiovascular [8] and central nervous system (CNS) [9] may also lead to death in some individuals, most notably in the elderly.

Much of the research related to SARS-CoV-2 has focused on the development of preventive vaccines. While this is certainly important to prevent future outbreaks, their effectiveness against the upcoming strains of SARS-CoV-2 that are likely to appear is hard to predict. Therefore, it is clear that additional strategies need to be put in place for the treatment of patients who have already acquired the infection, as the timely discovery of new drugs is not a viable strategy to confront the pressures imposed in the midst of a global pandemic situation. Therefore, the repurposing of existing drugs with an established safety profile to treat COVID-19 would be an interesting and more effective approach.

The use of IONPs as nanomaterials for clinical applications relies chiefly on their biocompatibility in moderate doses, the relatively well-known iron metabolic pathways, and their ability to be produced in a wide range of sizes and shapes with the potential for biofunctionalization [10]. IONPs have already passed through the preclinical stage to become a reality in clinical practice, currently mainly used in anti-anemic treatments and in magnetic resonance imaging (MRI) [11]. IONPs have also been successfully employed in a number of conditions as carriers of bioactive molecules, both in vitro and in animal models [12]. In the last few years, IONPs have also been demonstrated to be useful in clinical settings (i.e.,: clinical trials NCT01270139, NCT01436123 and NCT01927887) [13–15]. For example, in 2009 the rapid intravenous injection of ferumoxytol (Feraheme^®^) was shown to produce a significantly higher increase in hemoglobin than oral iron and it was also well tolerated by all patients [16]. The same year, ferumoxytol was approved by the Food and Drug Administration (FDA) and later, in 2012, by the European Medicines Agency (EMA) as an iron replacement therapy indicated for the treatment of iron deficiency anemia in adult patients with chronic kidney disease (CKD). Like Feraheme^®^, there are currently a large number of products to treat iron deficiency in patients with diseases such as CDK, of which the following stand out: Dexferrum^®^, high molecular weight iron dextran; Cosmofer^®^ and Infed^®^, low molecular weight iron dextran; Ferrlecit^®^, sodium ferric gluconate; Venofer^®^, iron sucrose; Ferinject^®^/Injectafer^®^, iron carboxymaltose; and Monofer^®^, iron isomaltoside 1000 [17]. The use of magnetic NPs is not only limited to therapy but also, they are widely used in diagnosis. For example, they can be employed as contrast agents in MRI, like the silicone-coated IONPs GastroMARK™ or Lumirem^®^ that received approval in 2001 and 2009, respectively [18]. Another milestone in the use of IONPs in oncology was the approval of Nanotherm^®^ in the European Union in 2010, aminosilane-coated IONPs designed to deliver hyperthermia to tumors [19].

The antiviral activity of metal and metal oxide NPs has been investigated to combat Dengue virus [20], rotavirus [21], influenza virus [22–24], human immunodeficiency virus 1 (HIV-1) [24] and SARS-CoV-2 [22, 25]. Plaque inhibition assays and quantitative real-time PCR (qRT-PCR) of viral transcripts in the presence of IONPs suggested these have antiviral activity against influenza [23]. Moreover, as a consequence of their ability to induce reactive oxygen species (ROS) production due to the metallic nature of its core, IONPs can catalyze lipid peroxidation of the viral lipid envelope and reduce the infectivity of influenza A viruses (subtypes H1N1, H5N1 and H7N9) [26]. Previous results from our group showed that IONP coatings could also contribute to ROS production in macrophages and tumor cell lines when IONPs are internalized by these cell types, with the IONPs coated with DMSA inducing more oxidative stress than the IONPs coated with APS [27], which would suggest that some coatings could provide a greater antiviral activity than others. However, despite their known therapeutic value, as far as we know IONPs have yet to be tested to treat SARS-CoV-2.

SARS-CoV-2 uses its S1-receptor binding domain (RBD) for its internalization and subsequent replication in the host cell, and a theoretical study showed how discrete Fe_2_O_3_ and Fe_3_O_4_ molecules can interact with this domain at the cell membrane. However, the model studied failed to address important issues, such as the size or the coating of the NP [28], parameters that determine their toxicity and therapeutic efficacy. Indeed, inorganic NPs have been of limited use in clinical practice due to the nanotoxicity associated with their size, shape, charge and surface chemistry [29]. For example, while generally considered a biocompatible material on a macroscale and as large NPs, gold NPs prove to be toxic at a size of 1.4 nm [30]. Therefore, specifically tailored metal and metal oxide NPs may offer interesting solutions to manage Coronaviridae infection and overcome the limitations of current methods for their treatment.

In this manuscript we present data showing that IONPs and Venofer^®^ (from now on FeraSpin™ R and Venofer^®^ will be referred to simply as FeraSpin R and Venofer) efficiently impair SARS-CoV-2 virus replication and transcription, as well as the production of infectious viruses in cultured cells, either when the cells were treated prior to or after infection. Moreover, we show that treating cells with IONPs and Venofer affects their oxidative stress and iron metabolism, which probably accounts for their beneficial effects on decreasing viral replication and production. Therefore, our results suggest that the IONPs and Venofer, may be used as a prophylactic and therapeutic treatment to combat SARS-CoV-2 infection.

## Materials and methods

### Synthesis of iron oxide and oxyhydroxide nanoparticles

Venofer^®^ (Vifor Pharma Ltd.) is a complex of polynuclear iron (III) oxyhydroxide in sucrose, also known as iron-carbohydrate complexes or iron sucrose, and it was developed to be administered as a medication to replace oral iron supplements to treat iron deficiency.

FeraSpin™ R (nanoPET-Pharma, Berlin, Germany) is a colloidal suspension of clustered 5 nm iron oxide magnetic NPs synthesized by aqueous coprecipitation of Fe (II) and Fe (III) in the presence of carboxydextran as a stabilizing agent, and it is used as a diagnostic imaging agent for in vivo MRI in mice (T2-weighted). The FeraSpin R colloidal suspension was purchased from nanoPET-Pharma (Berlin, Germany) and these particles have a hydrodynamic diameter of about 60 nm.

DMSA-IONP suspensions are aqueous colloids of an iron oxide magnetite core with a 10 or 16 nm diameter, as determined by Transmission Electron Microscopy (TEM, denominated as DMSA-IONP-10 and DMSA-IONP-16, respectively). The coated NPs were prepared by thermal decomposition of Fe(acac)_3_ in the presence of oleic acid (OA) as a surfactant and coated with DMSA by ligand exchange, as described previously [31, 32]. The biocompatible coating surrounding the iron plays a crucial role in stabilizing the iron core, slowing down the release of iron, protecting the particles from further aggregation, and sustaining the particles in a colloidal suspension that can be injected intravenously.

Magnetite NPs were prepared by decomposition of iron (III) acetylacetonate 99% (Acros Organics, Geel, Belgium) in benzyl ether (99%: Acros Organics, Geel, Belgium) in the presence of OA (80%: GPR Rectapur^®^, VWR, Leicestershire) and 1,2-dodecanediol 90% (ODA: Sigma Aldrich, San Luis, MO, USA). The concentration of the iron precursor was 0.1 M and the mixture was stirred at 100 rpm with nitrogen (9.5 L/min) introduced through the overhead stirrer guide for 1.5 h (the stirring and nitrogen flow were kept constant throughout the process). The reactor was then heated at an initial average temperature ramp of 3 ºC/min, left at 200 ºC for 2 h, and then heated again with a temperature ramp at a maximum power of 9 ºC/min until the mixture was boiling and refluxing at 286 ºC. Finally, the mixture was kept at the same temperature for 60 min to obtain 16 nm particles. Particles of 16 nm were obtained using a 1:3:2 molar ratio of Fe(acac)_3_:OA:ODA, while smaller particles of 10 nm were obtained by introducing oleylamine (OAM) together with the OA as a surfactant in a 1:3:3:2 molar ratio of Fe(acac)_3_:OA:OAM:ODA. The stirring was then stopped and the heating mantle removed in order to quench the reaction while maintaining the nitrogen flow. The product was washed three times with a mixture of toluene (99.5%: EssentQ^®^, Scharlau, Madrid, Spain) and ethanol (1:2 v/v), sonicated for 15 min and separated magnetically.

A ligand exchange reaction of OA for DMSA was used to transform hydrophobic magnetite NPs into hydrophilic ones. First, the particles were coagulated from the hydrophobic suspension (50 mg/5 ml) by adding ethanol, centrifuging (2825*g*, 10 min) and removing the solution. The particles were then dispersed in 20 ml of toluene and mixed with a solution of 100 mg of DMSA in 5 ml of DMSO (dimethyl sulfoxide), stirring the suspension obtained mechanically for 24 h. The supernatant was then discarded and the NPs coated with DMSA precipitated were successively mixed and centrifuged with ethanol several times to remove the free OA. The NPs were then dispersed in alkaline water, followed by redispersion at pH 7 and dialysis before the pH was adjusted to 7, and the NPs were filtered through a 0.2 μm pore size syringe.

APS-IONP-10 suspensions. The APS-coated nanoparticles were obtained by coprecipitation of a mixture of Fe(II) and Fe(III) salts in aqueous media, then coating the iron oxide core with APS [33]. Typically, to prepare particles with a 10 nm diameter a mixture of FeCl_3_.6H_2_O (0.09 mol) and FeCl_2_.4H_2_O (0.054 mol) in water (445 ml) was added slowly to 75 ml of NH_4_OH (25%) with vigorous stirring. The precipitate was washed three times with distilled water and recovered by magnetic decantation. The particles were then subjected to an acid treatment with 300 ml of HNO_3_ (2 M) under stirring for 15 min. Afterwards, the nitric acid was removed by magnetic decantation, and 75 ml of Fe (NO_3_)_3_ (1 M in water) and 130 ml of water were added to the particles. The mixture was then heated to boiling and stirred for 30 min. The particles were then cooled to room temperature (RT), and the supernatant was substituted with 300 ml of HNO_3_ (2 M) by magnetic decantation and stirred for 15 min. Finally, the particles were washed three times with water and redispersed in distilled water. The nanoparticle surface was coated with APS by adding 1.22 ml (0.005 mol) of APS dropwise to a mixture of 10 ml of particles (28 g Fe_2_O_3_ per liter of distilled water) and 10 ml of methanol with strong stirring. The mixture was left for 12 h and then the methanol was eliminated using a rotary evaporator. The APS-coated NP suspension was dialyzed, the pH was adjusted to 7 and the suspension was filtered through a 0.2 μm pore size syringe.

### Nanoparticle characterization

All the NPs obtained were characterized by TEM, X-ray diffraction (XRD), thermogravimetric (TG) analysis, Photon Correlation Spectroscopy (DLS), vibrating-sample magnetometry and inductively coupled plasma—optical emission spectrometry (ICP-OES). Particle size and shape were studied by TEM using a 100 keV JEOL microscope. TEM samples were prepared by placing one drop of a dilute suspension of NPs in water on a carbon coated copper grid and allowing the solvent to evaporate slowly at RT. The mean particle size and distribution was evaluated by measuring at least 250 particles.

**Table 1:** African Green monkey^a^ specific primers designed to study expression changes in Vero E6 cells

Gene (protein) NCBI reference sequence for the predicted mRNA	Forward (5′–3′)	Reverse (5′–3′)
Cat (Catalase) XM_008002350.2	AGAGAAATCCTCAGACACATC	CAACTTGAAAGTATGTGATCC
Sod1 (Superoxide Dismutase 1) XM_007965134.2	GAGCAGAAGGAAAGTAATGG	GATTAAAGTGAGGACCTGC
Sod2 (Superoxide Dismutase 2) XM_008007721.2	ATCATACCCTAATGATCTCAG	AGGACCTTATAGGATTTTCAG
Sod3 (Superoxide Dismutase 3) XM_038008646.1	CCTCCATTTGTACCGAAAC	GAAGATCGTCAGGTCGAAG
Duox1 (Dual Oxidase 1) XM_038009938.1	GTCATCAATCGGAACTCAAG	CAGAAATCCCGCACATCTTC
Duox2 (Dual Oxidase 2) XM_038009937.1	ATTTGAGGTGTCAGTGTTGG	TCACCCAGATGAAGTAGATCTT
Txndc2 (Thioredoxin domain-containing protein 2) XM_007974665.2	ATTATGAGGCATCTATGAAGG	CTTTCATTAGCATCACCTTCA
Txnrd2 (Thioredoxin reductase 2) NCBI Reference Sequence: XM_007975008.2	ACTTTAACATCAAAGCCAGC	GTAGCAATGATGATGTGGTCA
GAPDH (Glyceraldehyde 3-phosphate dehydrogenase) XM_037988380.1	TGCCATGGGTGGAATCATATTGGA	TCGGAGTCAACGGATTTGGGTCGT

^a^African Green monkey (*Chlorocebus sabaeus*, NCBI taxid 60711)

The phase of the iron oxide particles was identified by powder XRD. The x-ray patterns were collected between 10° and 80° (2θ) in a Bruker D8 Advance diffractometer with Cu Kα radiation. The crystal size was calculated from the broadening of the (311) reflection of the spinel structure following standard procedures. The presence of adsorbed anions on the particle surface was also investigated by TG analysis of the powders in a Seiko TG/ATD 320 U, SSC 5200. The analysis was performed between RT and 900 °C at a heating rate of 10 °C min^−1^ with an air flow.

Colloidal properties of the samples were studied in a Zetasizer Nano S apparatus (Malvern Instruments). The hydrodynamic size of the particles in suspension was measured by DLS and the electrophoretic mobility was measured as a function of pH at 25 ºC, using 10^−2^ M KNO_3_ as an electrolyte, and HNO_3_ and KOH to change the pH of the suspensions.

Magnetic characterization of the samples was carried out at RT in a vibrating sample magnetometer (MLVSM9 MagLab 9 T, Oxford Instruments). Magnetization curves were recorded by first saturating the sample in a field of 5 T and then, the saturation magnetization (Ms), and the coercive field (Hc) were determined for each sample. Ms values were evaluated by extrapolating the experimental results obtained in the high field range to infinite field, where magnetization linearly increases with 1/H. Samples were measured in powder form, pressed into a pellet. Iron leaching was analyzed by stirring the NP suspensions at 37 ºC for 24 h at 600 rpm in the presence of a buffer (pH 5 and pH 7) using an Eppendorf thermomixer comfort (Hamburg, Germany). Mixtures were then separated using Amicon^®^ Ultra Centrifugal filters and the iron content in the supernatant was determined by ICP-OES.

### Cell cultures

The African Green monkey kidney-derived epithelial Vero E6 cells were kindly provided by Prof. Luis Enjuanes (Centro Nacional de Biotecnología, CSIC, Spain). Vero E6 cells were grown in Dulbecco’s modified Eagle’s medium (DMEM: Gibco, Invitrogen, CA) supplemented with 25 mM HEPES and 10% fetal bovine serum (FBS: Fisher).

### Determination of the working dose of the different nanoparticles used

#### PrestoBlue assay

Cell viability was determined with the colorimetric PrestoBlue assay (Invitrogen). The PrestoBlue reagent containing a cell permeable blue and non-fluorescent solution of resazurin that can be metabolized inside cells to a red and fluorescent compound called resorufin. This change of colour and fluorescence serves as an indicator of cell viability. Vero E6 cells were seeded in a 96-well plate at a density of 3 × 10^4^ cells per well in a volume of 100 μl. After a 24 h incubation at 37 ºC, Vero E6 cells were treated with different concentrations of FAC, Venofer, FeraSpin R, APS-IONP-10, DMSA-IONP-10 or DMSA-IONP-16 (0–500 μg Fe/ml) for an additional 24 h. The PrestoBlue reagent was then added to each well, incubated in the same culture conditions for 2 h and the fluorescence was measured (560 nm excitation; 590 nm emission). Cell viability is indicated as a percentage of the fluorescence of treated cells relative to the fluorescence of the untreated cells.

#### TUNEL assays

TUNEL staining was performed to assay cell death. Vero E6 cells were seeded at a concentration of 4 × 10^4^ cells on coverslips previously situated inside a 24-well plate and they were incubated with two different concentrations of IONPs for 24 h. The concentrations tested were 50 μg Fe/ml and 250 μg Fe/ml for FAC, FeraSpin R, APS-IONP-10, DMSA-IONP-10 and DMSA-IONP-16, and 20 μg Fe/ml and 100 μg Fe/ml for Venofer. As a positive control of cell death in Vero E6 cells, the cells were incubated for 1 h with 2 mM H_2_O_2_. Images were taken with a dark-field Leica TCS SP5 confocal microscope with the 63X oil objective, and the total number of cells and TUNEL positive cells were analysed using ImageJ (NIH, USA) Software.

### Quantification of cellular iron uptake using an ICP-OES assay

Vero E6 cells were seeded in a 6-well plate at a density of 1 × 10^5^ cells per well and cultured for 24 h at 37 °C. The Vero E6 cells were then incubated with different nanoparticles in the same culture conditions for 3, 6 or 24 h: Venofer (20 or 100 μg Fe/ml), FeraSpin R (50 or 250 μg Fe/ml), APS-IONP-10 (50 or 250 μg Fe/ml), DMSA-IONP-10 (50 or 250 μg Fe/ml) or DMSA-IONP-16 (50 or 250 μg Fe/ml). In parallel, the amount of internalized iron ions was also analyzed after treating Vero E6 cells with FAC (50 or 250 µg Fe/ml) for the same times. Subsequently, the cells were washed three times with phosphate buffer saline (PBS) to remove the non-internalized NPs, harvested and counted in a Neubauer chamber. The samples were digested in HNO_3_ (1 ml) for 1 h at 90 °C and the amount of iron per cell was measured by ICP-OES (Perkin Elmer-2400).

### Cellular localization of IONPs visualized by TEM

For TEM microscopy, 2 × 10^6^ cells were seeded in petri dishes for 24 h, after which the optimal concentration for each type of treatment was added to the cells for 24 h: FAC 250 µg Fe/ml, Venofer 100 µg Fe/ml, FeraSpin R 250 µg Fe/ml, APS-IONP-10 250 µg Fe/ml, DMSA-IONP-10 250 µg Fe/ml, and DMSA-IONP-16 250 µg Fe/ml. Non-internalized IONPs were removed by washing with PBS, and the cells were then fixed at RT in 2% glutaraldehyde and 1% tanic acid diluted in 0.4 M HEPES (pH 7.2). The cells were washed and resuspended in HEPES buffer, post-fixed at 4 °C with 1% osmium tetroxide (1 h) and 2% uranyl acetate (30 min), dehydrated in a series of acetone solutions and gradually infiltrated with Epon resin. The resin was allowed to polymerize (60 °C, 48 h) and ultrathin Sects. (60–70 nm) were obtained with a diamond knife mounted on a Leica EM UC6 ultramicrotome. The sections were attached to a formvar/carbon-coated gold grid and visualized on a JEOL-1011 transmission electron microscope, acquiring images at different magnifications with a Gatan ES1000Ww camera.

### Viruses

SARS-CoV-2, isolated in Vero E6 cells and originating from a nasal swab from a patient infected in Madrid (Spain), was kindly provided by Prof. Luis Enjuanes (Centro Nacional de Biotecnología-CSIC, Spain).

### Viral infection and treatment of infected cells

To analyze the prophylactic effect of FAC, Venofer and the IONPs, confluent monolayers of Vero E6 cells (24-well plates) were treated for 24 h with the different IONPs at 50 and 250 µg Fe/ml (FeraSpin R, DMSA-IONP-10, APS-IONP-10 and DMSA-IONP-16), at 20 and 100 µg Fe/ml (Venofer), or at 50 and 250 µg Fe/ml (FAC). Cells were then infected with SARS-CoV-2 for 24 and 48 h (multiplicity of infection-MOI-0.001). The cell culture media was then collected, and titrated for 24 and 48 hpi. In addition, cells were collected at 6 and 16 hpi, and used for total RNA purification.

To analyze the therapeutic effect of FAC, Venofer and the IONPs, confluent monolayers of Vero E6 cells (24-well plates) were infected with SARS-CoV-2 (MOI 0.001), adding FAC, Venofer or the IONPs to the media at 1 hpi at the same concentrations used to study the prophylactic effect. The medium was collected at 24 and 48 hpi, and titrated. In addition, total RNA was purified from cells collected at 6 and 16 hpi.

### Viral titration

SARS-CoV-2 virus titrations were performed in Vero E6 cells grown in 24-well plates and infected with ten-fold serial dilutions of the virus. After 1 h absorption, the cells were overlaid with low electroendosmosis agarose (Pronadisa) and incubated for 3 days at 37 °C. The cells were then fixed with 10% formaldehyde in PBS and permeabilized with 20% methanol. Viral plaques were visualized and counted using crystal violet.

### Analysis of virus replication and transcription

Total RNA from untreated and FAC, Venofer and IONP-treated cells, either mock-infected or SARS-CoV-2-infected, were extracted using a total RNA extraction kit (Omega Bio-tek, GA, USA), according to the manufacturer’s instructions. Purified RNA (1 µg) was reverse-transcribed to cDNA using a High-Capacity cDNA Reverse-Transcription kit (Applied Biosystems) and random primers. To analyze the effect of the treatments with FAC, Venofer, and IONPs on viral replication, qRT-PCR was performed using the primers SARS-2-RdRp-15431-VS (5′GTGAAATGGTCATGTGTGGCGG-3′) and SARS-2RdRp-15530-RS (5′-CAAATGTTAAAAACACTATTAGCATA-3′), complementary to the viral genomic RNA (gRNA) [34]. To analyze the effect of FAC, Venofer and IONPs on viral transcription, the primers, SARS-2-leader-VS: 5′-TCCCAGGTAACAAACCAACCAACT, complementary to the leader sequence; and SARS-2-7a-RS: 5′-AAATGGTGAATTGCCCTCGT-3, were used to amplify the gene 7 subgenomic (sg) mRNA by qRT-PCR [35]. In all the cases, GAPDH was used to normalize the data, amplified using the 5′-TGCCATGGGTGGAATCATATTGGA-3′ (sense) and 5′-TCGGAGTCAACGGATTTGGGTCGT-3 (antisense) primers, and quantified using the threshold cycle (2^−ΔΔ*CT*^) method [36].

### Analysis of the induction of oxidative stress as a result of IONP treatment

#### Dihydrorhodamine 123 (DHR) staining

The production of ROS was quantified by dark-field confocal staining with the DHR probe (Molecular probes, Carlsbad, CA, USA), a non-fluorescent ROS indicator that can be oxidized inside cells to the fluorescent rhodamine 123. Vero E6 cells were cultured on coverslips in a 24-well plate for 24 h and once they were attached to the coverslip, the medium was removed and the cells were treated for 24 h with a 250 μg Fe/ml (FAC, FeraSpin R, APS-IONP-10, DMSA-IONP-10 and DMSA-IONP-16) or 100 μg Fe/ml (Venofer) suspension. As a positive control of oxidative stress, the Vero E6 cells were incubated for 1 h with 1 mM H_2_O_2_. After incubation, the coverslips were rinsed three times with PBS and the cells were incubated for 30 min with DHR (diluted 1:500 in medium) under cell culture conditions. After washing the cells again three times with PBS, they were fixed for 15 min with paraformaldehyde (PFA) 4%, stained for 10 min with DAPI (diluted 1:500 in PBS), washed and mounted with Fluoromont-G. Images were taken under a dark-field Leica TCS SP5 confocal microscope with the 63X oil objective. For dark-field acquisition of IONPs, the 488 nm laser light was used, and the images were analysed and the DHR signal intensity was quantified with Image J software.

#### Measurement of glutathione (GSH)

The GSH in the cells was assessed with a colorimetric kit (GSH Assay kit, ab239727, Abcam). GSH, a tripeptide that contains thiol groups, can act as an electron donor or acceptor in cells, being one of the cell’s main antioxidant resources. Vero E6 cells were plated in a 100 mm diameter culture dish and then treated with a 250 μg Fe/ml (FAC, FeraSpin R, APS-IONP-10, DMSA-IONP-10 and DMSA-IONP-16) or 100 μg Fe/ml (Venofer) suspension for 24 h in the presence or absence of the ROS scavenger N-acetylcysteine (NAC, 200 μM). The cells were then collected and the protein concentrations were determined with a BCA assay (Pierce™ Rapid Gold BCA Protein Assay Kit: Thermo Scientific™). The GSH detection kit used contains a chromophore that after reduction by GSH produces a stable product that can be measured at 450 nm, such that the absorbance is proportional to the amount GSH in the sample. The GSH concentration is calculated relative to the absorbance with a GSH standard provided in the kit and expressed in nmol/mg of protein.

#### Analysis of the expression of transcripts of genes involved in the antioxidant response by qRT-PCR after IONP treatment

Vero E6 cells treated for 24 h with a 250 μg Fe/ml (FAC, FeraSpin R, APS-IONP-10, DMSA-IONP-10 and DMSA-IONP-16) or a 100 μg Fe/ml (Venofer) suspension were collected, and the total RNA from untreated and treated cells was extracted using the High Pure RNA Isolation Kit (Roche). The RNA was quantified with NanoDrop, and 2 μg of RNA was reverse-transcribed to cDNA using the High-Capacity cDNA Reverse Transcriptase kit (Applied Biosystems, Thermofisher) and random primers. The cDNA was used to perform qRT-PCR with Power SYBR Green PCR mix (Applied Biosystems, Thermofisher) and primers designed specifically to amplify *Chlorocebus sabaeus* transcripts of catalase (Cat), superoxide dismutase 1, 2, and 3 (Sod1, Sod2, Sod3), dual oxidases 1 and 2 (Duox1, Duox2), thioredoxin domain-containing protein 2 (Txndc2) and thioredoxin reductase 2 (Txnrd2) genes (see Table 1) according to the *Chlorocebus sabaeus* predicted mRNA sequences (NCBI taxonomy number 60711). Primers were synthesized by Sigma-Aldrich. The qRT-PCR data was analysed by the threshold cycle (2^−ΔΔ*CT*^) method [36] and normalized to GAPDH expression.

### Analysis of the influence of oxidative stress on SARS-CoV-2 replication

Confluent monolayers of Vero E6 cells (24-well plates) were treated for 24 h with NAC (200 µM), a recognized ROS scavenger [37], or left untreated as a control. The cells were then infected with SARS-CoV-2 (MOI, 0.001) and the extracellular medium containing the virus was replaced at 1 hpi with a suspension of the IONPs and NAC (200 µM), or with a suspension of the IONPs without NAC as a control. The medium was then collected at 48 hpi and the virus titrated.

### Analysis of the changes induced in iron metabolism genes as a consequence of the internalization of IONP and of viral infection

To analyse the effect of NP treatment on genes involved in iron metabolism, Vero E6 cells treated for 24 h with a 250 μg Fe/ml (FAC, FeraSpin R, APS-IONP-10, DMSA-IONP-10 and DMSA-IONP-16) or a 100 μg Fe/ml (Venofer) suspension were collected. In addition, cells were infected with SARS-CoV-2 and 1 hpi, they were treated with the same concentrations of FAC, Venofer and IONPs for a further 24 h. The total RNA from untreated and treated cells was extracted with the High Pure RNA Isolation Kit (Roche), quantified using NanoDrop and 2 μg of RNA was reverse-transcribed to cDNA using the High-Capacity cDNA Reverse Transcriptase kit (Applied Biosystems, Thermofisher) and random primers. The cDNA was used to perform qRT-PCR with a Power SYBR Green PCR mix (Applied Biosystems, Thermofisher) and primers designed specifically to amplify transcripts for Transferrin Receptor (TFRC), SLC11A2 (encoding divalent metal transporter 1, DMT1), SLC48A1 (encoding heme transporter 1, HRG1), SLC40A1 (encoding ferroportin, FPN1), iron responsive element binding protein 2 (IREB2) and lipocalin 2 (LCN2, encoding neutrophil gelatinase-associated lipocalin-NGAL) in Vero E6 cells according to the *Chlorocebus sabaeus* predicted mRNA sequences (NCBI taxonomy number 60711, see Table 2). Primers were synthesized by Sigma-Aldrich. The qRT-PCR data was analysed by the threshold cycle (2^−ΔΔ*CT*^) method [36] and normalized to GAPDH expression.

**Table 2:** African Green monkey^a^ primers designed to study changes in gene transcripts in Vero E6 cells

Gene (protein) NCBI reference sequence for the predicted mRNA	Forward (5′–3′)	Reverse (5′–3′)
TFRC (Transferrin receptor protein1) XM_008009680.2	AAGATTCAGGTCAAAGACAG	CTTACTATACGCCACATAACC
SLC11A2 (Divalent metal transporter 1-DMT1) XM_037997121.1	GAGTATGTTACAGTGAAACCC	GACTTGACTAAGGCAGAATG
SLC48A1 (Heme transporter 1-HRG1) XM_008003003.2	ATGTACATGCAAGATTACTGGAG	GTCTGTGAGGCTCTGATG
SLC40A1 (Ferroportin—FPN1) XM_007965607.2	AAAGATACTGAGCCAAAACC	GTTGTAGTAGGAGACCCATC
IREB2 (Iron Responsive element-binding protein 2) XM_038010228.1	CATTTTCCGTCAGGACAGAC	CCAGTAAATATGGTCCTTTG
LCN2 (Neutrophil gelatinase-associated lipocalin—NGAL) XM_008006094.2	GGAAAGAGAAGTGTGACTACTG	GTGATTCTGAATGTTGCCCAG
GAPDH (Glyceraldehyde 3-phosphate dehydrogenase) XM_037988380.1	TGCCATGGGTGGAATCATATTGGA	TCGGAGTCAACGGATTTGGGTCGT

^a^African Green monkey (*Chlorocebus sabaeus*, NCBI taxid 60711)

### Visualization of Vero E6 cells infected with SARS-CoV-2 and treated with DMSA-IONP-10 by TEM

For TEM, Vero E6 cell monolayers were either infected with SARS-CoV-2 (MOI 0.001) and at 1 hpi treated with DMSA-IONP-10 for an additional 24 h (therapeutic treatment), or the cells were treated with DMSA-IONP-10 NPs for 24 h and then infected with the virus for 24 h (prophylactic treatment). The cells were then fixed for 1 h in situ with 2% glutaraldehyde in phosphate Na/K buffer (pH 7.4) at RT, removed from the dishes and transferred to Eppendorf tubes. After centrifugation, the cells were washed three times in phosphate Na/K buffer (pH 7.4) and processed for embedding in epoxy TAAB 812 resin (TAAB Laboratories, Berkshire, England) according to standard procedures. The cells were treated with a mixture of 1% osmium tetroxide and 0.8% potassium ferricyanide in distilled water for 1 h at 4 °C and after five washes with distilled water, the samples were incubated with 2% uranyl acetate in water for 1 h, washed three times and dehydrated twice in increasing concentrations of acetone (50, 70, 90 and 100%) for 10 min each at RT. Resin infiltration was accomplished in increasing concentrations of acetone-Epon (3:1, 1:1, 1:3 and 100% Epon) and it was polymerized at 60 °C for 2 days. Ultrathin sections of the cells were stained with saturated uranyl acetate and lead citrate, and examined at 80 kV in a Jeol JEM-1010 (Tokyo, Japan) electron microscope.

### Statistical analysis

All the data are presented as the mean ± standard deviation (SD) and analyzed by a one-way and two-way analysis of variance (ANOVA). A Student’s, Sidak and Tukey test were applied to calculate the differences between the distinct values. Values of p < 0.05 were considered statistically significant: *p < 0.05, **p < 0.01, ***p < 0.001, and ****p < 0.0001. GraphPad Prims version 6.1 was used for all the statistical analyses.

## Results and discussion

### Description and characterization of the different nanoparticles used

**Fig. 1:**
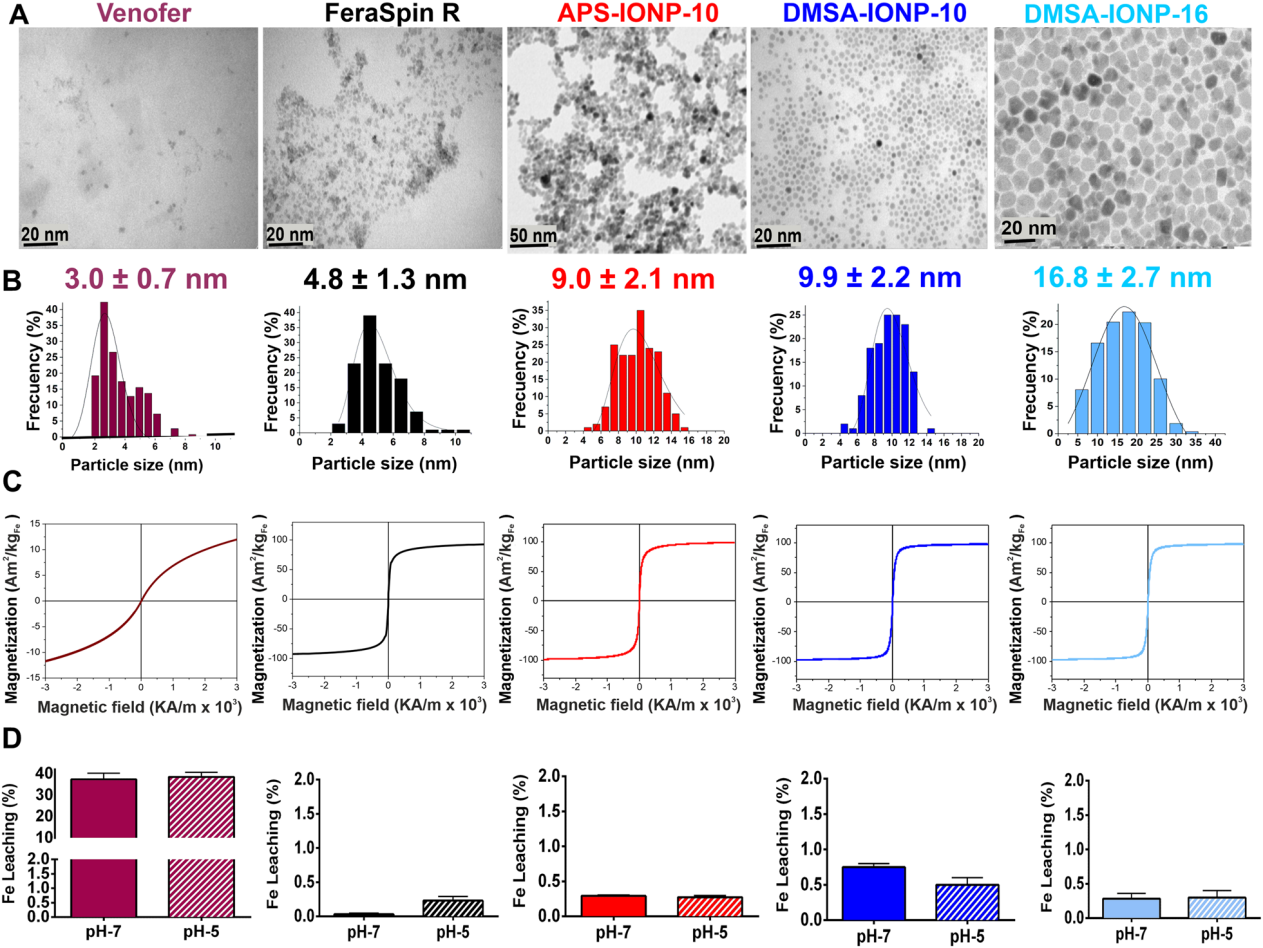
Physicochemical characterization of the different nanoparticles used. **A** TEM images of the iron oxide nanoparticles used, scale bar: 20–50 nm. **B** Nanoparticle size distribution and Gaussian fitting. **C** Magnetization curve at RT for the IONPs showing their superparamagnetic behavior. **D** Data related to iron leaching from the iron oxide under the specified conditions (pH 7 or 5), depending on the size and on the type of coating

Different formulations based on coated IONPs and IOHNPs have been tested for their ability to treat and/or prevent viral infections. One such commercial option that was designed and approved as a clinical treatment for anemia in humans is Venofer (Vifor Pharma Spain), while FeraSpin R (nanoPET Pharma GmbH) is a contrast agent used for MRI in small animals. In addition, three further samples based on uniform magnetic IONPs were prepared for this study: two of them were produced by thermal decomposition in organic medium and coated with DMSA to analyze the effect of particle size, namely DMSA-IONP-10 (10 nm in diameter) and DMSA-IONP-16 (16 nm in diameter); and the other produced by co-precipitation (10 nm in diameter) and coated with APS to study the effect of the coating, APS-IONP-10 (Fig. 1A).

Venofer contains 3 nm diameter antiferromagnetic NPs (as measured by TEM: Fig. 1 A and B, and Table 3) that are sucrose-coated to a hydrodynamic size of around 45 nm (Table 3). XRD of Venofer (also named iron sucrose) shows the nanometric particles are most likely composed of two-line ferrihydrite with a mean crystal size of 3.8 nm [38]. Iron oxyhydroxides like ferrihydrite, are weakly ferromagnetic at RT, with a saturation magnetization of 18 Am^2^/kg (Table 3).

**Table 3:** Physicochemical characterization of IOHNPs and IONPs

Sample	Coating	Particle size TEM (nm)	Crystal size X-ray (nm)	Hydrodynamic size and PDI (nm)	Z-Potential (mV)	MS at RT (Am2/KgFe)
Venofer	Sucrose	3.0 ± 0.7	3.8	45 (0.21)	− 10	18
FeraSpin R	Carboxydextran	4.8 ± 1.3	5	66 (0.24)	− 36	94
APS-IONP-10	3-Aminopropyl-triethoxysilane	9.0 ± 2.1	8	104.7 (0.26)	+ 19	102
DMSA-IONP-10	Dimercaptosuccinic acid	9.9 ± 2.2	10	65 (0.25)	− 33	100
DMSA-IONP-16	Dimercaptosuccinic acid	16.8 ± 2.7	13	74 (0.23)	− 27	99

*PDI* degree of polydispersity

FeraSpin R is a suspension of superparamagnetic IONPs of around 4.8 nm in diameter (Fig. 1A and B and Table 3) and their carboxydextran coating gives them a hydrodynamic diameter of about 66 nm [39]. X-ray diffractograms of FeraSpin R and IONP samples have peaks assigned to a spinel structure similar to magnetite or maghemite. The crystal sizes (see Table 3) calculated from the (311) peak broadening agree well with the TEM size (Fig. 1A and B). Magnetite or maghemite NPs are ferrimagnetic and they present saturation magnetization values at RT of 50–120 Am^2^/kgFe (Fig. 1C and Table 3), increasing with particle diameter. In all cases, the magnetic behavior is superparamagnetic at RT, with a reversible hysteresis loop (zero remanence) that assures negligible aggregation in the absence of a magnetic field.

The different NP coatings affect their hydrodynamic size and surface charge, which is of particular importance in nanomedicine as it dictates the NP-cell interactions, their biodistribution and their degradation [27, 39, 40]. In all cases, hydrodynamic size is around or below 200 nm and their polydispersity index (PDI) ≤ 0.26, these parameters reflecting the absolute size of the particles in solution, including the coating and hydration layer. The coatings on the NP surface, such as sucrose, carboxydextran or DMSA, account for the negative surface charge at a neutral pH (i.e.: between − 10 and − 36 mV: Table 3). However, positive surface charges are obtained (+ 19 mV: Table 3) when the NPs are coated with amines like APS. The mass percentage of the coating varied between 10 and 35% from the largest (16 nm) to the smallest particles (5 nm: Additional file 1: Fig. S1A and B), as determined through thermogravimetric analyses (Additional file 1: Fig. S1C).

Iron solubility and release (Fig. 1D) are important parameters of anti-anemic NPs, and these might be of interest to understand the potential antiviral effect of the NPs. As such, NP dissolution was evaluated by analyzing the iron leaching after 24 h at temperatures simulating body temperature (37 °C) to, and at pH 5 to mimic intralysosomal conditions or pH 7 to mimic cytosolic conditions. The highest dissolution rate was measured for Venofer (~ 38%), reflecting its hydroxide nature and the fact it contains the smallest particles [41]. Oxide particles of similar size coated with DMSA dissolved less in similar conditions (< 5%: Additional file 1: Fig. S1D) and among the remaining samples the dissolution decreased from 0.7% to 0.3% as the IONP size increased from 10 to 16 nm. For the same particle size, dissolution of the particles increased with the coating as follows: carboxydextran < APS < DMSA.

### Determining the cytotoxicity of IONPs, IOHNPs and FAC (ferric ammonium citrate) in Vero E6 cells

**Fig. 2:**
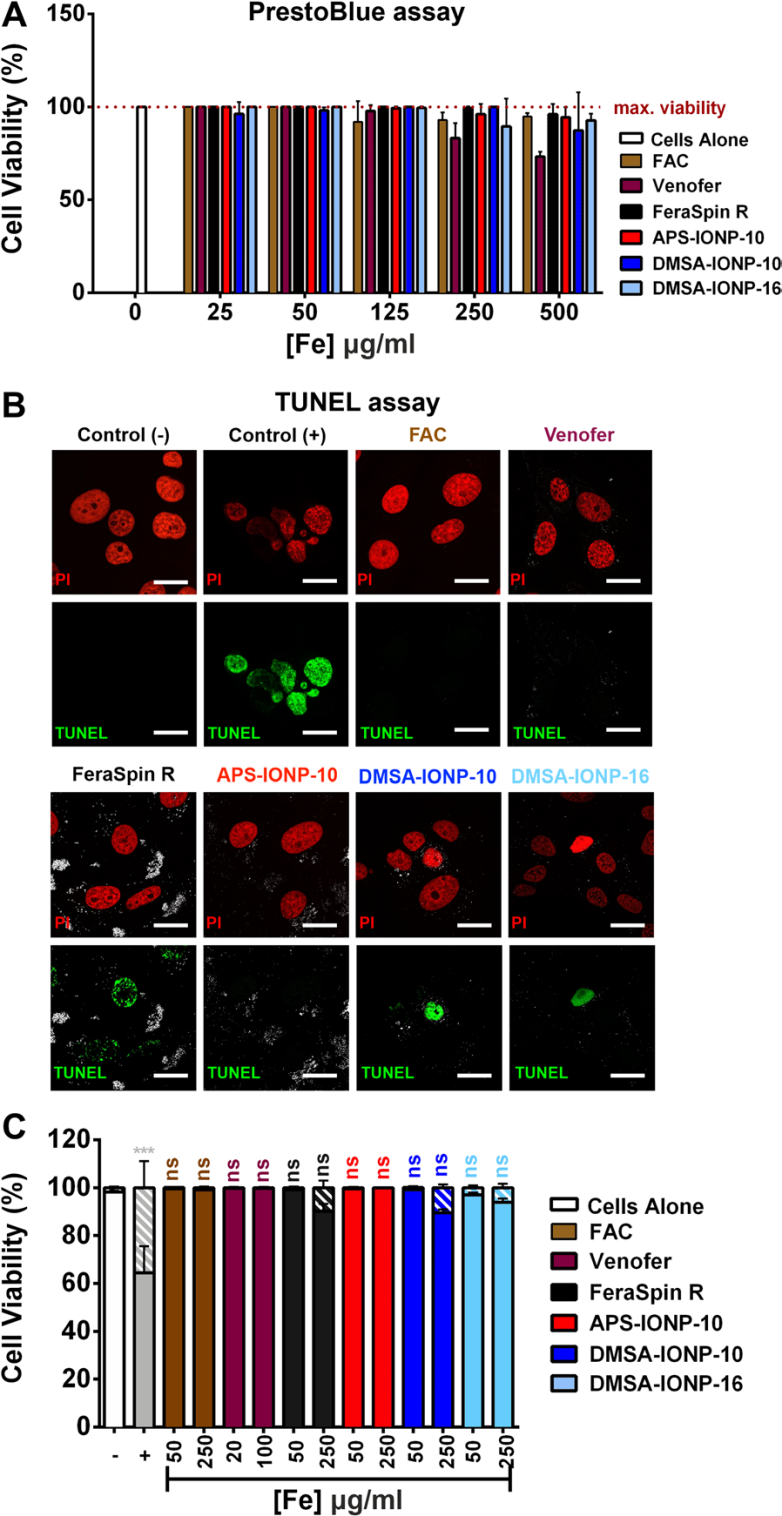
Evaluation of FAC, Venofer and IONP toxicity, and quantification of dead Vero E6 cells. **A** Viability of Vero E6 cells after treatment with FAC, Venofer or IONPs, as measured with the PrestoBlue fluorometric test. **B** and **C** TUNEL staining analysis of the proportion of dead Vero E6 cells after incubation with FAC, Venofer and IONPs. Images were taken with a 63X oil objective under a 3X zoom: TUNEL positive cells (green), PI counterstaining (red) and nanoparticles (grey). The positive control cells (+) in **B** and **C** were cells treated for 1 h with H_2_O_2_ (2 mM) and the results (mean ± SD) are representative of three independent experiments. One-way analysis of variance (ANOVA) and a Student’s t-test were used to assess the TUNEL data, and the asterisks indicate significant differences: *p < 0.05, **p < 0.01 and ***p < 0.001

To study the possible antiviral effect of FAC, Venofer and the other IONP formulations used, we first determined the possible working concentration of all the formulations in Vero E6 cells using the PrestoBlue assay to indirectly measure mitochondrial metabolism. The viability of the cultured Vero E6 cells was assayed after exposure for 24 h to different concentrations of FAC, Venofer or the four distinct types of IONP (from 0 to 500 μg Fe/ml). FAC, FeraSpin R, APS-IONP-10, DMSA-IONP-10 and DMSA-IONP-16 were not toxic to Vero E6 cells (> 90% viability) even at concentrations as high as 250 μg Fe/ml (Fig. 2A), whereas cell viability was reduced to 77% following exposure to Venofer at 250 μg Fe/ml (Fig. 2A). While DMSA-IONP-10 produced some cytotoxicity at higher concentrations (500 μg Fe/ml), reducing Vero E6 viability to 70%, FAC, FeraSpin R, APS-IONP-10 and DMSA-IONP-16 still did not decrease Vero E6 cell viability below 90% (Fig. 2A). Therefore, we decided on using a working concentration of 250 µg Fe/ml for FAC, FeraSpin R, APS-IONP-10, DMSA-IONP-10 and DMSA-IONP-16, and of 100 μg Fe/ml for Venofer. However, in addition to this optimal high concentration (100 or 250 μg Fe/ml) where Vero E6 cell viability was above 90%, we selected a second lower working concentrations (20 or 50 μg Fe/ml) where Vero E6 cell viability was 100% to analyze whether the different treatments show any dose-dependent effects.

In addition to the assay of mitochondrial metabolism, the induction of apoptosis in Vero E6 cells was studied. Cells were exposed to two different concentrations of the NPs (50 and 250 μg Fe/ml for FAC and the IONPs, and 20 and 100 μg Fe/ml for Venofer) and then assayed by TUNEL (Fig. 2B and C). From the images and quantification of the TUNEL positive Vero E6 cells, no significant differences in cell viability were evident at either of the concentrations tested for all the formulations (Fig. 2B and C). Only FeraSpin R, DMSA-IONP-10 and DMSA-IONP-16 produced a slight reduction in cell viability at the highest dose of 250 μg Fe/ml, with 9.7%, 10.6% and 6.1% of the cells in the culture not viable, respectively (Fig. 2C).

### FAC, IOHNP and IONP uptake by Vero E6 cells

**Fig. 3:**
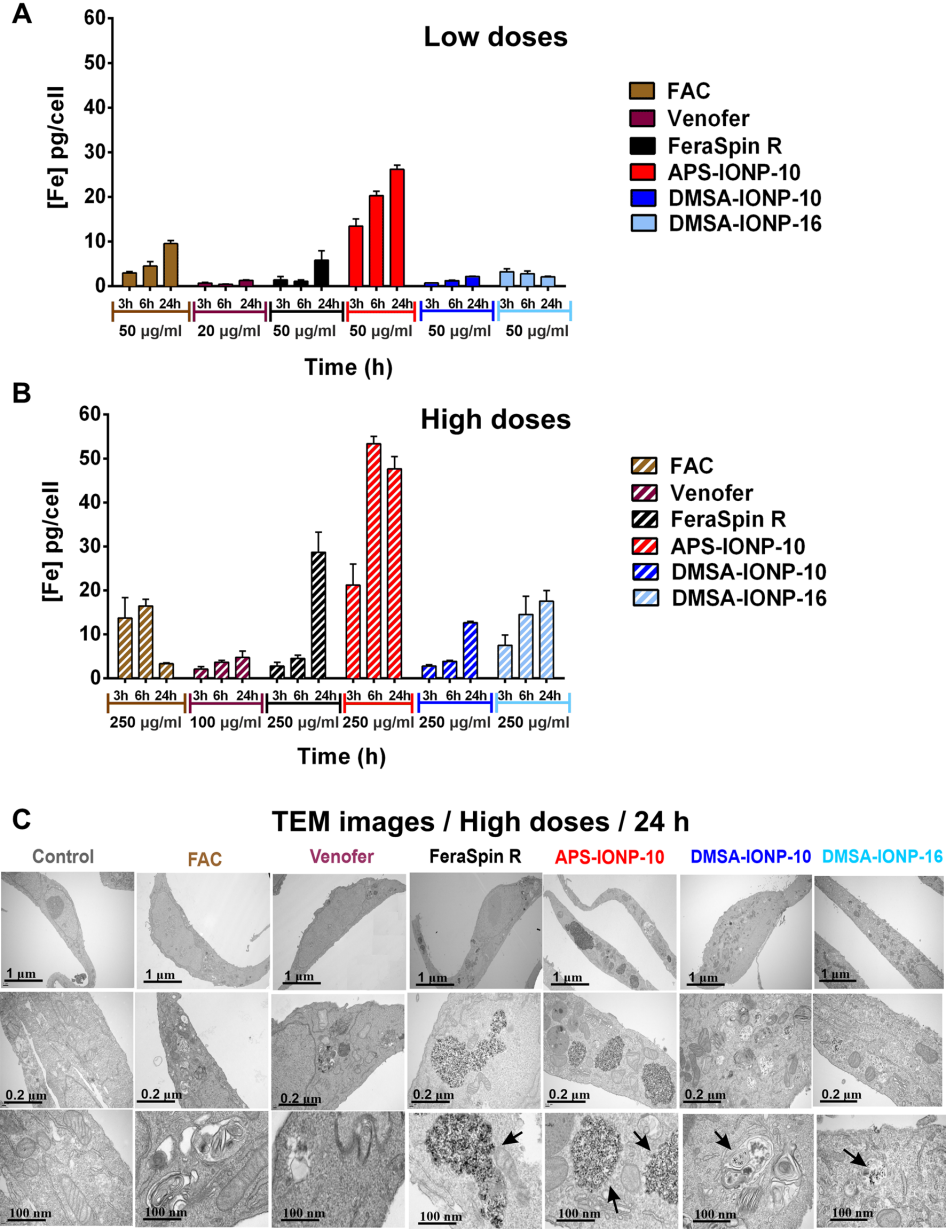
FAC, Venofer and IONP uptake and localization in Vero E6 cells. **A** and **B** Evaluation of FAC, Venofer, FeraSpinR, APS-IONP-10, DMSA-IONP-10 and DMSA-IONP-16 internalization by Vero E6 cells after a 3, 6 and 24 h incubation at lower (20 or 50 μgFe/ml) (**A**) and higher (100 or 250 μgFe/ml) concentrations (**B**), as measured by ICP-OES. The graph shows the increase in iron content in Vero E6 cells over basal levels (untreated cells). The data (mean ± SD) are representative of three independent experiments. **C** Representative TEM images of Vero E6 cells after treatment with IONPs. High detail images showing the presence of IONPs in vesicles within the Vero E6 cells: scale bar 1 μm–100 nm. The vesicles containing the nanoparticles are indicated with arrows in the lower magnification images. Scale bar: 100 nm

Vero E6 cells were incubated for 3, 6 or 24 h with different concentrations of FAC (the iron salt used as control, 50 or 250 μg Fe/ml), Venofer (20 or 100 μg Fe/ml) or IONPs (50 or 250 μg Fe/ml), and the IONPs internalized by the cells was assessed by measuring the intracellular iron concentration of the cultured cells by ICP-OES: Fig. 3A and B. As expected, dose-dependent iron uptake was evident in the cells treated with FAC, Venofer and IONPs (Fig. 3A and B). The maximum iron uptake by Vero E6 cells incubated with FAC (250 μg/ml) was observed at 6 h (16.45 pg Fe/cell), the iron concentration decreasing at 24 h (3.3 pg Fe/cell). However, when the cells were treated with the lowest dose of FAC the maximum iron uptake was observed after 24 h (10 pg/cell). In the case of Venofer and FeraSpin R, maximum iron uptake by Vero E6 cells was observed at 24 h for both the lower and higher doses, with more iron taken up when the cells were treated with the highest dose (4.8 pg Fe/cell for Venofer and 28.7 pg Fe/cell for FeraSpin R). Of the different IONPs analyzed, the highest uptake was detected with APS-IONP-10, reaching a maximum at 6 h in the cells treated with the higher dose (53.3 pg Fe/cell). Finally, less internalization of the DMSA-coated IONPs was observed relative to the other IONPs (APS-IONP-10 and FeraSpin R), with maximal ion uptake at 24 h: 12.6 pg Fe/cell DMSA-IONP-10 internalization; 17.5 pg Fe/cell DMSA-IONP-16 internalization at the higher doses (Fig. 3B). These results indicate that IONPs are internalized distinctly according to their coating, suggesting that the endocytotic pathways by which Vero E6 cells take up these particles differ depending on the type of IONP.

The quantification of the iron internalized by Vero E6 cells when treated with lower doses (20 or 50 μg Fe/ml) showed that for all IONPs, internalization was mainly dependent on the dose used, as the amount of iron internalized was greater when the cells were treated with the highest dose in all cases (100 or 250 μg Fe/ml). However, FAC and Venofer seem to produce other kinetics of iron entry and exit, perhaps due to the availability of iron in ionic form.

The subcellular localization of IONPs was analyzed by TEM after a 24 h incubation, showing that all types of IONPs and Venofer entered the Vero E6 cells, and accumulated in cytoplasmic vesicles (Fig. 3C). However, the amount of particles that accumulated in the vesicles and their compaction differed depending on the type of IONP. A correlation was observed between the quantification of the IONPs internalized and the TEM images, with greater compaction of APS-IONP-10 inside endocytic vesicles, followed by FeraSpin R and the DMSA-IONPs. In TEM images we observed less contrast of the Venofer particles than IONPs because their core is composed of an iron oxyhydroxide. In the case of FAC treatment, an iron salt, we did not observe any signal because the iron is present as ions which meant it was not evident within vesicles.

### The effect of FAC, IOHNPs and IONPs on viral production and replication

**Fig. 4:**
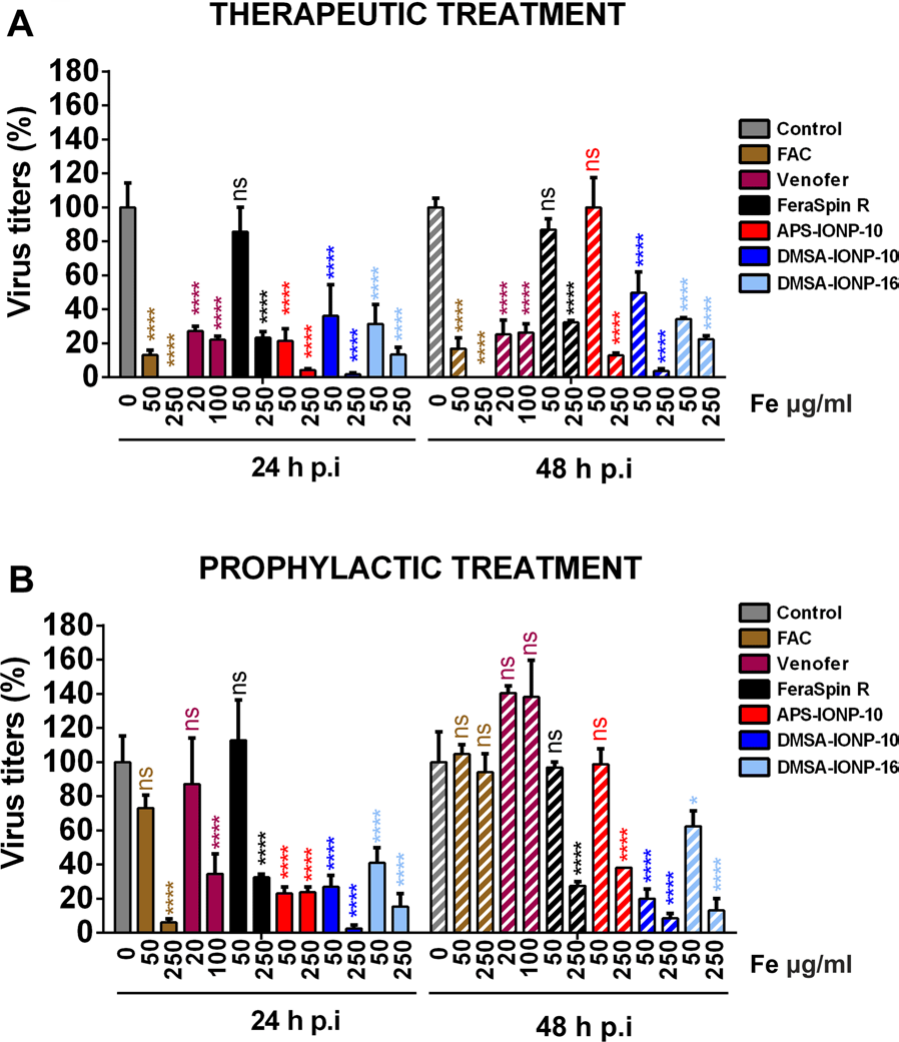
Post-treatment and pre-treatment of cells with FAC, Venofer and IONPs reduces SARS-CoV-2 production. **A** Vero E6 cells were infected with SARS-CoV-2 and 1 hpi, the cells were treated with FAC, Venofer or the IONPs (FeraSpin R, DMSA-IONP-10, APS-IONP-10, and DMSA-IONP-16) at two different concentrations, or left untreated (control cells). **B** Vero E6 cells were treated with FAC, Venofer or the IONPs indicated in A, and 24 h after treatment, the cells were infected with SARS-CoV-2. **A** and **B** Cell culture supernatants were collected at 24 and 48 h post-infection (hpi) and the viral titer was assessed with a plaque assay. Viral titers were determined and represented relative to the titers in control, untreated cells (%). The data (mean ± SD) are representative of three independent experiments and they were analyzed by two-way ANOVA followed by a Sidak’s multiple comparison test: *p < 0.05; **p < 0.01; ***p < 0.001; ****p < 0.0001; and ns, no significant differences

To analyze whether iron in the form of FAC, IOHNPs (Venofer) or IONPs (FeraSpin R, DMSA-IONP-10, APS-IONP-10 and DMSA-IONP-16) affects SARS-CoV-2 production, cells were treated with each 24 h before infection (prophylactic effect) or 1 h after infection (therapeutic effect), given that the virus is estimated to enter the cell in the first 10 min of exposure [42]. Viral titers were measured in the cell culture supernatants at different times post-infection and interestingly, FAC, Venofer and IONPs had a dose-dependent effect against the virus production, both therapeutically and prophylactically (Fig. 4A and B). In terms of the therapeutic effect, the highest, non-cytotoxic concentrations of FAC, Venofer and the IONPs decreased SARS-CoV-2 production at 24, and 48 h post-infection (hpi), with FAC, DMSA-IONP-10 and APS-IONP-10 reducing the viral titers most strongly. In fact, the highest concentrations (250 µg Fe/ml) of FAC, DMSA-IONP-10 and APS-IONP-10 reduced the viral titers at 24 hpi by 99.98, 98.0 and 95.50%, respectively, and at 48 hpi by 99.99, 96.25 and 87.50%, respectively (Fig. 4A). For the prophylactic effect, the highest concentrations of all the treatments reduced the viral titers at 24 hpi, whereas FAC and Venofer did not affect the viral titers at 48 hpi relative to the control, untreated cells. The maximum reduction of the viral titers in cells that were first treated with the IONPs, Venofer or FAC and then infected, were observed at the highest concentrations of FAC, DMSA-IONP-10, and DMSA-IONP-16, which reduced viral titers at 24 hpi by 94.0, 98.0 and 85.0%, respectively (Fig. 4B). Interestingly, while the viral titers were not reduced at 48 hpi when cells were treated with the highest concentration of FAC, at this time point DMSA-IONP-10 and DMSA-IONP16 did reduce the viral titers by 92 and 87%, respectively (Fig. 4B). Hence, therapeutic and prophylactic treatment of cells with FAC, Venofer and IONPs diminished the production of SARS-CoV-2 infectious viruses, although to differing extents.

**Fig. 5:**
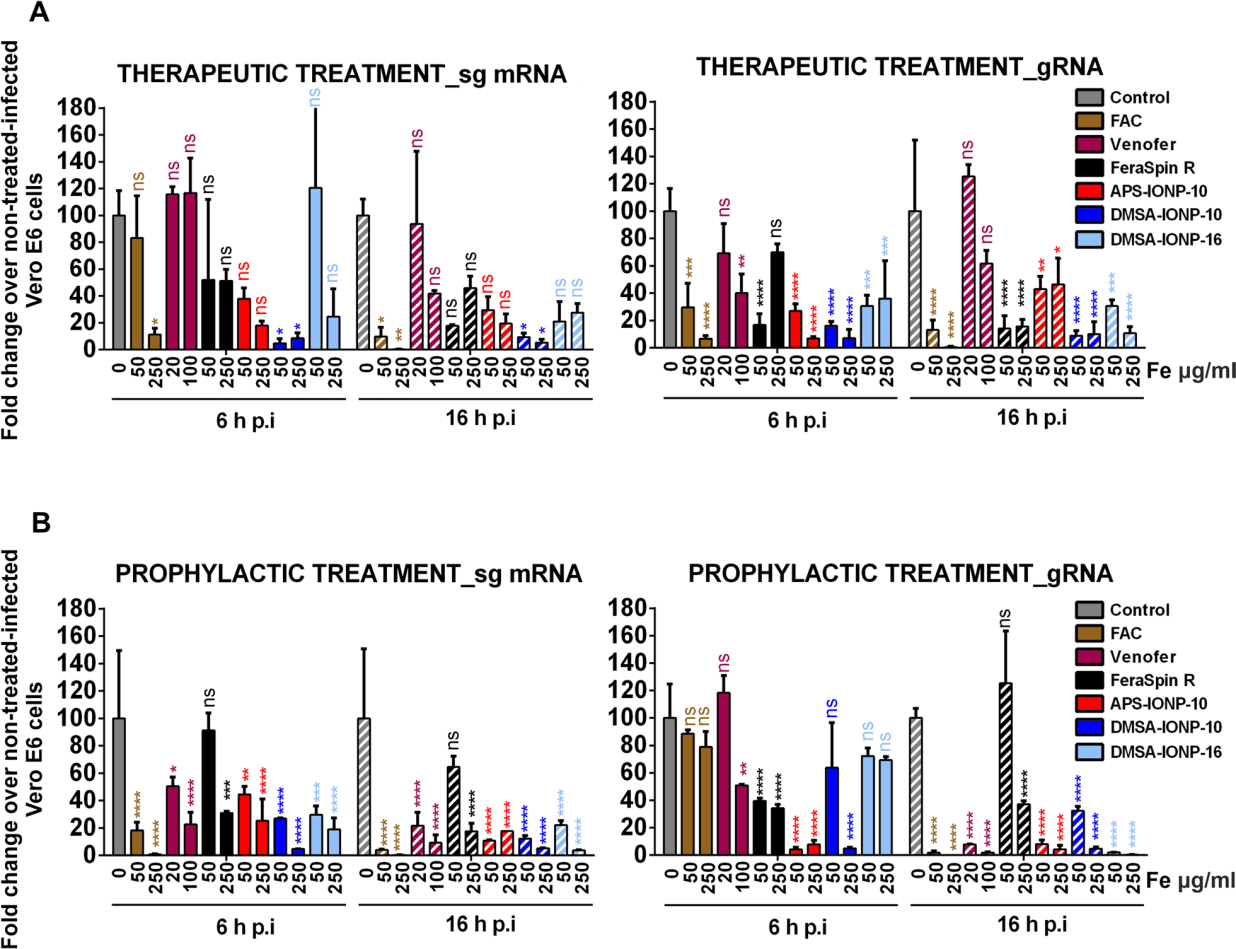
Pre-treatment and post-treatment of cells with FAC, Venofer and IONPs reduces SARS-CoV-2 replication and transcription. **A** Vero E6 cells were infected with SARS-CoV-2 and 1 hpi, the cells were treated with FAC, Venofer or the IONPs (FeraSpinR, DMSA-IONP-10, APS-IONP-10, and DMSA-IONP-16) at two different concentrations, or left untreated (control cells). **B** Vero E6 cells were treated with FAC, Venofer or the IONPs indicated in A, and 24 h after treatment, the cells were infected with SARS-CoV-2. **A** and **B** Total RNA was extracted at 6 and 16 hpi, and the levels of viral gene 7 sg mRNA, viral gRNA and GAPDH RNA were measured by qRT-PCR. The levels of gene 7 sg mRNA and viral gRNA were normalized to the levels of GAPDH, and represented relative to the levels in control, untreated cells (%). The data (mean ± SD) were representative of three independent experiments and they were evaluated by two-way ANOVA followed by a Sidak’s multiple comparison test: *p < 0.05; **p < 0.01; ***p < 0.001; ****p < 0.0001; and ns, no significant differences

To analyze whether the treatment of cells with FAC, Venofer and IONPs directly affects viral replication and/or transcription, cells were treated with FAC, Venofer and IONPs before or after SARS-CoV-2 infection, and the amounts of gRNA were measured by qRT-PCR at 6 and 16 hpi to analyze viral replication, and the amounts of gene 7 sg mRNA were measured to analyze viral transcription. The therapeutic treatment of cells after SARS-CoV-2 infection with the highest doses of FAC and IONPs decreased the amount of gene 7 sg mRNA at 6 and 16 hpi, while treating the cells with Venofer only decreased gene 7 sg mRNA at 16 hpi. Remarkably, gRNA levels decreased significantly after treatment with FAC and all IONPs at 6 and 16 hpi, at both doses. In the case of Venofer treatment, a significant reduction in gRNA levels was only observed 6 hpi in cells treated with the highest dose **(**Fig. 5A).

The prophylactic treatment of cells prior to SARS-CoV-2 infection (treatment) with the highest doses of FAC, Venofer and IONPs decreased the amount of gene 7 sg mRNA at 6 and 16 hpi. In terms of gRNA levels, we observed a reduction at 16 hpi with all treatments at the higher doses, whereas no reduction in gRNA was evident at 6 hpi with FAC and DMSA-IONP-16 (Fig. 5B). These results strongly suggest that treating cells with FAC, Venofer or IONPs affects viral replication and transcription, although to different extents depending on the therapeutic agent.

**Fig. 6:**
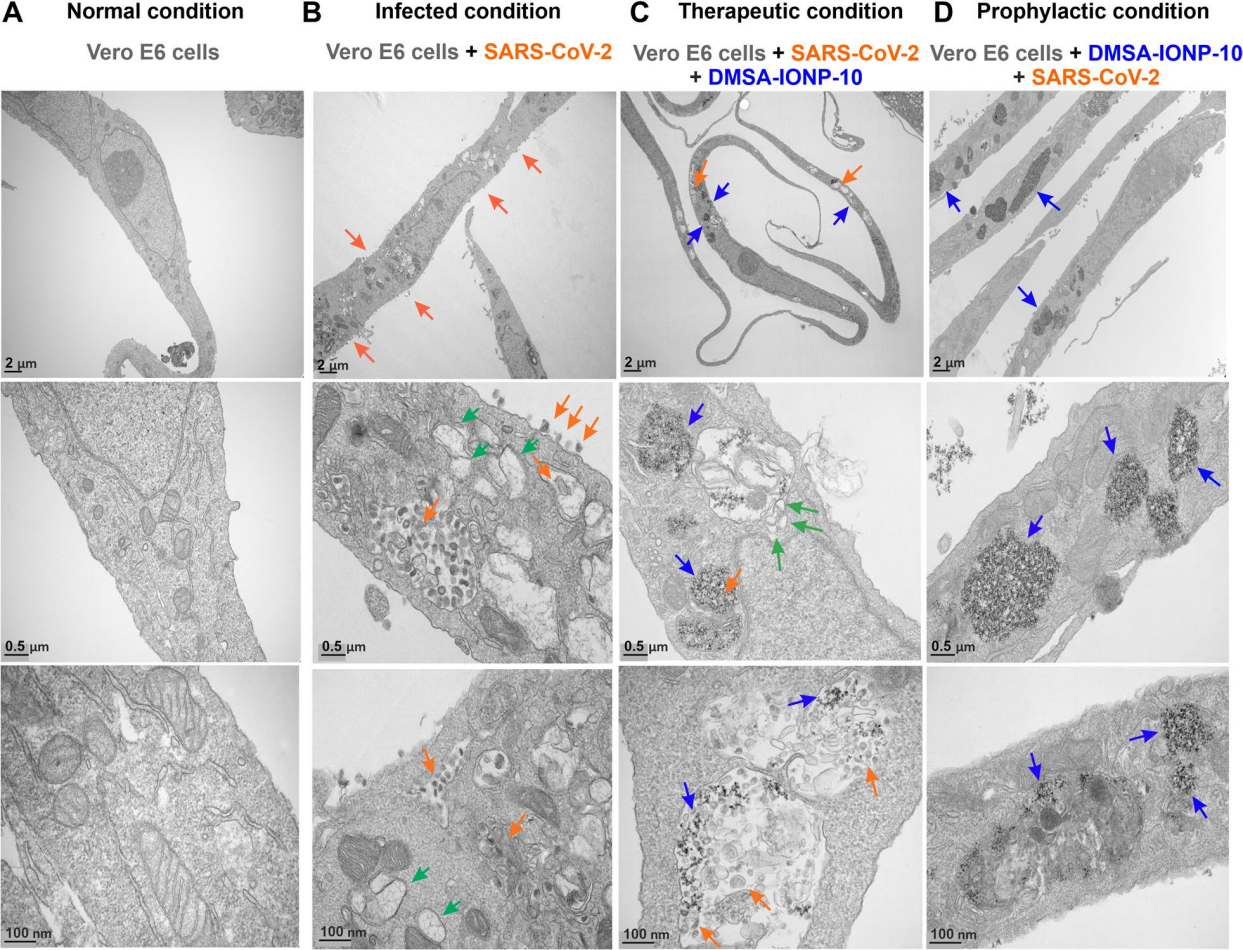
Ultrastructural analysis of infected cells treated with DMSA-IONP-10. **A** Normal condition: untreated and mock-infected Vero E6 cells (Control). **B** Infected condition: Vero E6 cells were infected at a MOI of 0.001 with SARS-CoV-2 for 24 h. **C** and **D** The cells were infected with SARS-CoV-2 and 1 hpi the cells were treated with DMSA-IONPs-10 at 250 μg Fe/ml (**C**) or alternatively, the cells were treated with DMSA-IONP-10 at 250 μg Fe/ml and then infected (**D**). In all cases the cells were processed for TEM of ultrathin sections at 24 hpi. Colored arrows indicate the presence of viral particles (in orange), DMVs (in green) and the accumulation of DMSA-IONP-10 inside the Vero E6 cells (in blue). Scale bars: 2 μm, 0.5 μm, and 100 nm, as indicated

To analyze whether structural changes to SARS-CoV-2-infected cells when treated with IONPs could in part explain their antiviral activity, Vero E6 cells infected with SARS-CoV-2 and treated with Venofer or the different IONPs were analyzed by TEM. Cells were either infected with SARS-CoV-2 and 1 hpi treated with Venofer or the different IONPs for a further 24 h (therapeutic treatment), or they were treated with Venofer or the IONPs for 24 h and then infected for 24 h (prophylactic treatment), and processed for TEM analysis. Fewer intracellular and extracellular viral particles were evident in the cells treated with Venofer or the different IONPs either before or after infection than in the untreated control cells (Fig. 6 for DMSA-IONP-10, and Additional file 1: Fig. S2 for Venofer, FeraSpin R, APS-IONP-10 and DMSA-IONP-16), which was correlated to the lower amounts of infectious viruses detected in the cell culture supernatants of Venofer and IONP-treated cells (Fig. 4). As the best prophylactic and therapeutic antiviral activity was observed with DMSA-IONP-10, we focused on larger TEM images of infected cells treated with these particles to better explain the observed structural effects (Fig. 6). Interestingly, large double-membrane vesicles (DMVs), considered to be the organelles for coronavirus replication and transcription [43, 44], were observed in untreated, infected cells, and in cells that were first infected and then treated with DMSA-IONP-10. However, DMVs were hardly seen in the cells that were first treated with DMSA-IONP-10 and then infected (Fig. 6D). Furthermore, IONP internalization was enhanced in the cells that were first treated with DMSA-IONP-10 rather than when infected cells were then exposed to DMSA-IONP-10 (Fig. 6B), suggesting that viral infection impairs DMSA-IONP-10 internalization to some extent. These data indicate that the pre-treatment of cells with DMSA-IONP-10 impaired the formation of DMVs (Fig. 6D), providing an explanation for the decreased viral replication and transcription observed in the cells after exposure to DMSA-IONP-10 (Fig. 5). The structural effects observed cells treated with Venofer, FeraSpin R, APS-IONP-10 or DMSA-IONP-16 (Additional file 1: Fig. S2) were less evident as with the DMSA-IONP-10 treatment, where the viral particles and DMVs seem to disappear (Fig. 6D).

### Induction of oxidative stress as a consequence of FAC, IOHNP or IONP uptake and how the antiviral activity of these agents is influenced by their effect on oxidative stress

**Fig. 7:**
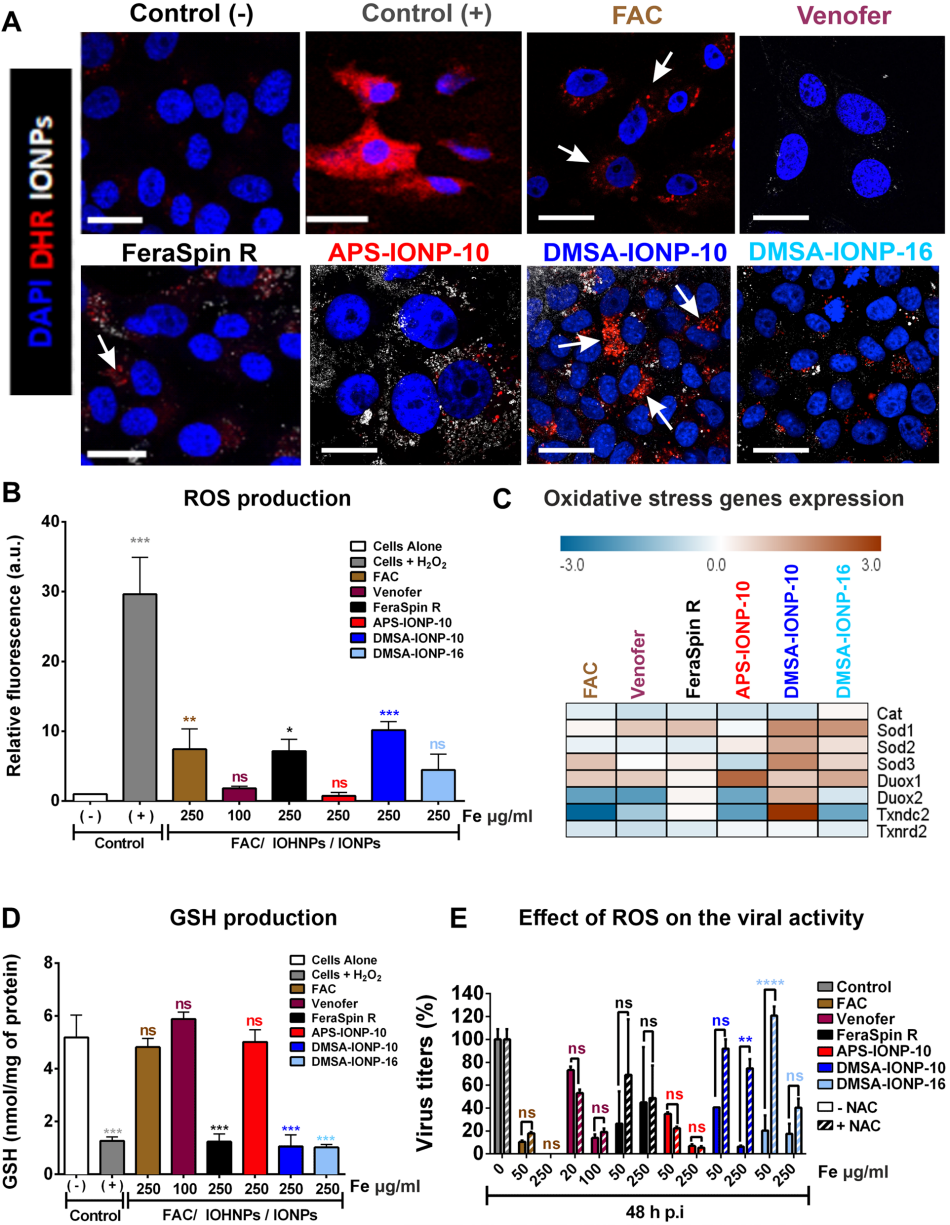
Oxidative stress and ROS induction in cells after treatment with FAC, Venofer or IONPs. **A** and **B** ROS generation observed by DHR fluorescence and quantitative image analysis of DHR fluorescence intensity using Image J software: Control (−), untreated Vero E6 cells; and control (+), Vero E6 cells incubated with 1 mM H_2_O_2_. Images were taken with a 63X oil objective under a 3X zoom. **C** Quantification of oxidative stress gene expression by qRT-PCR (mRNA levels) in Vero E6 cells after treatment with FAC, Venofer or the different IONPs (Venofer, FeraSpin R, APS-IONP-10, DMSA-IONP-10 or DMSA-IONP-16). The expression was compared to that in untreated cells and the data were normalized to the expression of GAPDH. **D** Glutathione content in untreated [Control (−)] Vero E6 cells, and in Vero E6 cells incubated for 24 h with FAC, Venofer or IONPs, or with 1 mM H_2_O_2_ [Control (+)]. **E** Effect of ROS on the antiviral activity of FAC, Venofer or the IONPs. Confluent monolayers of Vero E6 cells were treated for 24 h with N-acetylcysteine (NAC) or left untreated as a control. The cells were then infected with SARS-CoV-2 (MOI, 0.001) and 1 hpi, the extracellular medium containing the virus was replaced with a suspension of FAC, Venofer and IONPs, together with NAC (200 µM), or with a suspension of the FAC, Venofer or IONPs without NAC as a control. The medium was collected from the cells and titrated at 48 hpi. The data are shown as mean ± SD (n = 3) and analyzed with a two-way analysis of variance (ANOVA) and Tukey’s multiple comparisons test: *p < 0.05, **p < 0.01, ***p < 0.001

IONPs [40, 45–48] and FAC [49, 50] induce ROS production and trigger oxidative stress in different cell types. Poly (aniline-co-pyrrole) nanocomplexes have been seen to also regulate intracellular ROS levels and supress influenza A viral infection [51], and 200 nm Fe_3_O_4_ NPs can dampen the viral infectivity of different influenza A virus subtypes (H1N1, H5N1, and H7N9), affecting viral lipid envelope integrity [26]. To determine whether the oxidative stress induced by FAC, Venofer or the different IONPs could be in part responsible for the antiviral effect seen in Vero E6-infected cells, their capacity to produce ROS in Vero E6 cells was studied. Vero E6 cells were treated with FAC (250 μg Fe/ml), Venofer (100 μg Fe/ml) or the different IONPs (250 μg Fe/ml) and after a 24 h incubation, the cells were stained with the DHR (dihydrorhodamine 123) probe, a non-fluorescent probe that fluoresces when oxidized by ROS inside cells. DHR staining showed that FAC, Venofer and all IONPs produced ROS, albeit to a different extent (Fig. 7A). The different sizes and coatings of IONPs affected the capacity to produce ROS in cells distinctly, with DMSA-IONP-10 the most oxidative IONP that produced a ten-fold change in DHR fluorescence relative to the control (1 a.u. of fluorescence), followed by FAC, FeraSpin R and DMSA-IONP-16 (7.4 a.u, 7.2 a.u. and 4.5 a.u. fluorescence, respectively). In contrast, Venofer and APS-IONP-10 were the IONPs with the least oxidative potential, producing similar levels of ROS to the controls (Fig. 7B).

Cells have different mechanisms to neutralize the exacerbated production of ROS in order to maintain redox homeostasis, one of which is the oxidation of thiol groups on cysteine residues of proteins, such as thioredoxin and GSH [52]. In addition, cells encode different antioxidant enzymes like Cat, Sod, Duox, peroxiredoxins and thioredoxin [53]. To further study the induction of oxidative stress in Vero E6 cells, the antioxidant response induced by IONPs was assessed. The expression of gene transcripts involved in the antioxidant response was studied by qRT-PCR (Fig. 7C): Cat; Sod 1, 2 and 3; Duox 1 and 2; and thioredoxin reductases (*Txndc2* and *Txnrd2*). The results of this study showed that DMSA-IONP-10 and DMSA-IONP-16 were capable of inducing the strongest expression of the different antioxidant genes (Fig. 7C).

In addition, the total amount of GSH was measured in Vero E6 cells treated with FAC, Venofer and the different IONPs, both in the presence or absence of the antioxidant NAC (Additional file 1: Fig. S3A). First, we determined the working concentration of NAC in Vero E6 cells with the PrestoBlue assay, which indirectly measures mitochondrial metabolism. Vero E6 cells were incubated for 24 h with different concentrations of NAC (from 0 to 500 μM) and the viability of the cultured cells was measured. As the viability of the cells was higher than 95% when the cells were incubated with NAC up to 250 μM, decreasing to 70% when the cells were incubated with NAC at 500 μM (Additional file 1: Fig. S3A), we used a concentration of 200 μM for the following experiments. Then, we verified that the addition of the ROS inhibitor (NAC) did not affect the internalization of FAC, Venofer and the IONPs, as measured by an ICP-OES assay (Additional file 1: Fig. S3B**)**. GSH has a thiol group capable of being oxidized when there is an increase of ROS in the cells and thus, decreasing the total GSH in cells correlates with increased ROS production and oxidative stress [54]. IONPs with a greater capacity to produce ROS (FeraSpin R, DMSA-IONP-10 and DMSA-IONP-16) reduced the total amount of GSH in cells to a higher extend (Fig. 7D). By contrast, treating cells with Venofer and APS-IONP-10, the IONPs producing the least ROS did not diminish the GSH in the cells (Fig. 7D). Although FAC treatment produced high amounts of ROS in cells (Fig. 7A and B), it did not produce a reduction in GSH levels (Fig. 7D), indicating that glutathione is not the main antioxidant machinery activated to reduce the oxidative stress induced by FAC, yet it would trigger the activation of Sod 3 due to the strong expression of this gene induced by FAC (Fig. 7C). Together, these results suggest that FeraSpin R, DMSA-IONP-10 and DMSA-IONP-16 can induce significant oxidative stress in Vero E6 cells, activating different antioxidant machineries, being DMSA-IONP-10 and DMSA-IONP-16 those that induced the highest levels of oxidative stress when measured by the three approaches used: ROS production (Fig. 7A and B), the induction of antioxidant enzymes (Fig. 7C), and GSH levels (Fig. 7D).

To analyze whether the oxidative stress induced by FAC, Venofer or IONPs is responsible for their antiviral effects, cells were infected and at 1 hpi, the cells were treated with the FAC, Venofer or IONPs together with NAC, a ROS scavenger [37]. Viral titers were measured at 48 hpi and to verify that NAC dampens ROS production, a DHR analysis was performed in the presence and absence of NAC (Additional file 1: Fig. S4). As seen previously (Fig. 7B), treating the cells with the highest doses of DMSA-IONP-10, followed by FAC, FeraSpin R and DMSA-IONP-16, induced ROS production (Additional file 1: Fig. S4), whereas Venofer and APS-IONP-10 did not induce ROS production in these conditions. When treated with DMSA-IONP-10, FAC, FeraSpin R or DMSA-IONP-16 together with NAC, the ROS produced was similar to that observed in the control, untreated cells (Additional file 1: Fig. S4), indicating that NAC decreases ROS production after DMSA-IONP-10, FAC, FeraSpin R and DMSA-IONP-16 treatments. Interestingly, viral titers were similar in control cells not exposed to FAC, Venofer or IONPs, or not treated with NAC (Fig. 7E). However, in cells exposed to DMSA-IONP-10 or DMSA-IONP-16, viral titers increased when the cells were treated with NAC relative to cells that remained untreated in the original medium. Importantly, the treatment of NAC did not significantly, affect the internalization of these IONPs, indicating that the effect of NAC on viral titers is not due to changes in the internalization of the IONPs (Additional file 1: Fig. S3B). This effect was not observed in the cells exposed to FAC, Venofer, FeraSpin R or APS-IONP-10 (Fig. 7E). These results suggested that the induction of oxidative stress in cells by DMSA-IONP-10 and DMSA-IONP-16 was at least partially responsible for the antiviral effect of these IONPs. However, this was not the case for FAC, Venofer and APS-IONP-10, probably due to the fact that these compounds do not induce oxidative stress in the cells as efficiently as DMSA-IONP-10 and DMSA-IONP-16, as measured by three different approaches (Fig. 7B–D).

Regarding the correlation of the ROS generated by NPs and the antiviral effect observed, FeraSpin R, DMSA-IONP-10 and DMSA-IONP-16 are the NPs inducing the highest levels of oxidative stress (Fig. 7A, B and D), and when Vero E6 are treated with these IONPs, a reduction in viral titers was observed (Fig. 4). However, Venofer and APS-IONP-10, which do not seem to induce oxidative stress, also cause significant decreases in viral titers (Fig. 4). The main difference observed between the different NPs in terms of oxidative stress induction is that the decreases in viral titers seems to be maintained over time in the IONPs that induce a greater amount of ROS, while for NPs that do not cause oxidative stress the effect on viral titers seems to be reversible over time, mainly in the case of Venofer after prophylactic treatment (Fig. 4B). These results suggest that the induction of oxidative stress could enhance the effect of the NPs in reducing viral titers, but it is not the only mechanism that causes this reduction in virus production.

### Changes in the expression of gene transcripts involved in iron metabolism as a consequence of FAC, IOHNP and IONP internalization, and as a consequence of SARS-CoV-2 infection

**Fig. 8:**
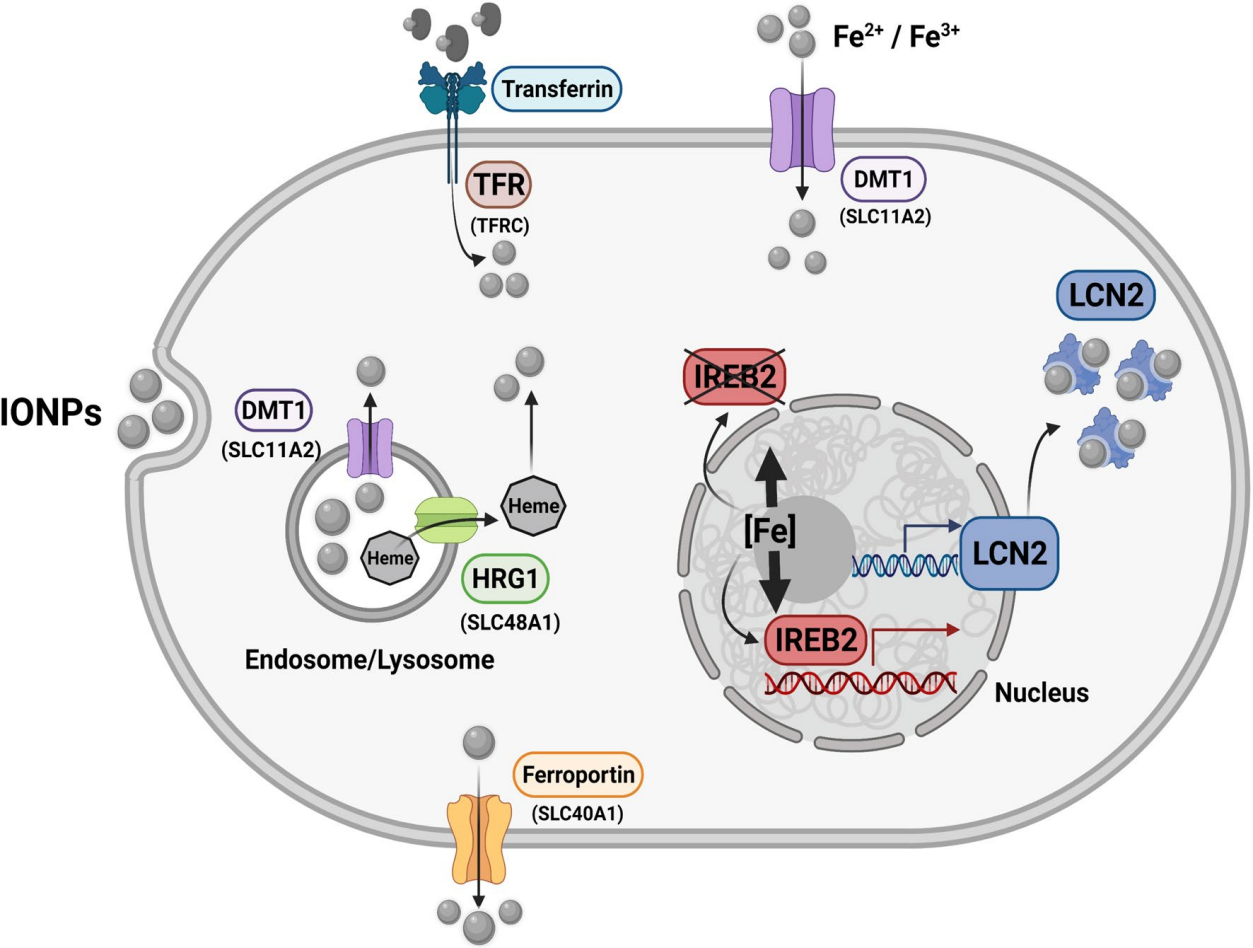
Schematic representation of the regulation of endogenous iron metabolism. Iron acquisition is dependent on endocytosis of diferric transferrin via the transferrin receptor (TFRC). In acidified endosomes, iron is freed from transferrin and exported into the cytoplasm by DMT1. In the cytosol, excess iron is sequestered within heteropolymers of ferritin H and L chains. Cellular iron efflux is mediated by ferroportin (FPN1) and requires iron oxidation on the extracellular side [56]. HRG1 is another protein that is localized in acidified vesicles and it serves to export heme groups stored in these compartments to the cytosol [57]. When iron levels in the cytoplasm are high, lipocalin (LCN2), in coordination with siderophores as co-factors, interacts with iron and forms a ternary complex [58]. Iron homeostasis is regulated by iron responsive proteins, such as IREB2, by binding to iron-responsive elements (IREs). When iron is limited, IREB2 binds to the IREs of some iron metabolism genes that repress ferritin and ferroportin translation, and that stabilize DMT1 and TFRC mRNA. By contrast, when iron is found in the cell, IREB2 degradation is induced and thus, it cannot bind to IREs and induce ferritin or ferroportin expression, whereas DMT1 and TFRC degradation is induced [59]

We previously reported that treating different cell types with IONPs induces changes in the expression of different genes involved in iron metabolism [45, 46, 48] and thus, we assessed whether the treatment of Vero E6 cells with IONPs also affects the expression of such genes. This could be of particular interest since it is accepted that viruses generally require enhanced cell metabolism to prosper, as well as high levels of iron to replicate [55]. Hence, we wondered whether IONPs might inhibit SARS-CoV-2 infection by affecting the transcription of genes involved in iron metabolism. To assess whether FAC, Venofer or IONPs altered genes involved in iron metabolism or transport, total RNA was extracted from the cells after exposing them to these agents for 24 h and the expression of specific genes was analysed by qRT-PCR: SLC11A2, DMT1; SLC40A1, or FPN1; TFRC; IREB2; SLC48A1, or HRG-1; and LCN2 or NGAL. In terms of their physiological activity, TFRC or TFR allow iron ions attached to transferrin to enter the cell, while DMT1 also encodes a protein that facilitates the entry of iron into the cell but that also moves iron ions from endosomal compartments to the cytosol. HRG1 is also located in endosomal compartments and it has been implicated in the translocation of iron to the cytosol, while FPN1 exports iron from the cell and IREB2 is a transcription factor involved in the control of iron metabolism. Finally, LCN2, that encodes NGAL, attaches to and sequester iron in cytosol and therefore diminishes the total cytoplasmic iron available (Fig. 8).

**Fig. 9:**
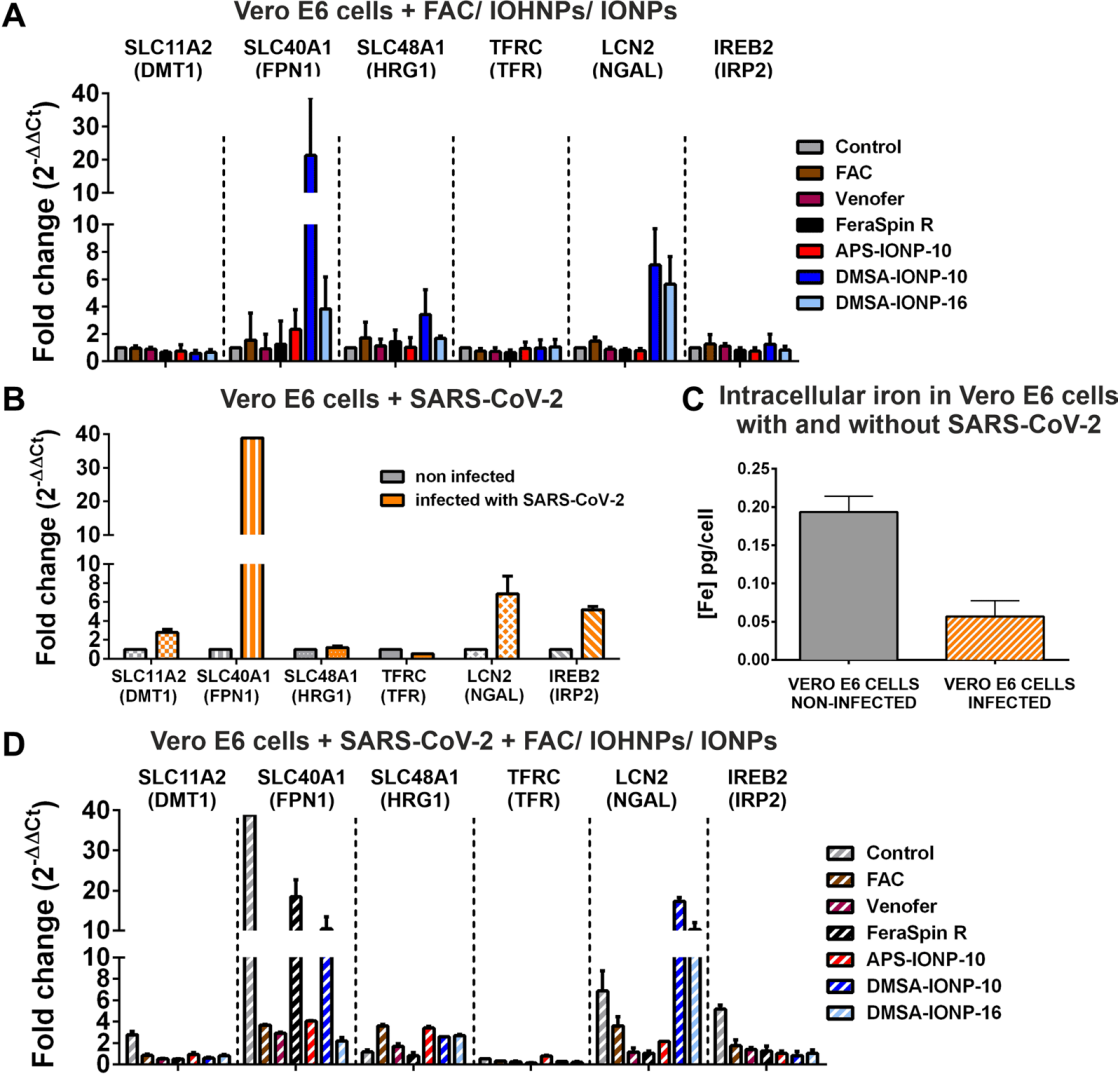
FAC, Venofer and IONP treatments alter the iron metabolism in Vero E6 cells. **A** The effect of treatment with FAC, Venofer or IONPs on the expression of genes involved in iron metabolism. **B** The effect of SARS-CoV-2 infection on genes involved in iron metabolism. **C** The concentration of intracellular iron in non-infected and SARS-CoV-2-infected cells was measured by ICP-OES. **D** The effect of FAC, Venofer or IONP treatment on genes involved in iron metabolism in SARS-CoV-2-infected cells. Confluent monolayers of Vero E6 cells were infected with SARS-CoV-2 (MOI, 0.001) and at 1 hpi, the extracellular medium containing the virus was replaced with a suspension of FAC, Venofer or IONPs . Total RNA was purified at 24 hpi, and the expression of SLC11A2, SLC40A1, SLC48A1, TFRC, LCN2 and IREB2 was analyzed by qRT-PCR and normalized to the GAPDH expression in each sample. Data shown as the mean ± SD (n = 5)

Interestingly, DMSA-IONP-10 and DMSA-IONP-16 were the IONPs that induced the strongest changes in iron metabolism related genes, enhancing the expression of HRG1 that moves iron to the cytoplasm by three- and two-fold, respectively (Fig. 9A), increasing the expression of LCN2 by seven- and six-fold, respectively (Fig. 9A), and augmenting the expression of FPN1 by 21- and four-fold, respectively (Fig. 9A). As a result, these IONPs appear to hijack the total free-iron in the cytoplasm and induce strong iron export from the cell, likely decreasing the ionic iron bioavailability. As DMSA-IONP-10 and DMSA-IONP-16 are the IONPs that most strongly induce LCN2 and FPN1 (Fig. 9A), thereby significantly diminishing iron bioavailability, and prophylactically these IONPs are those that most impair virus replication (Fig. 4B), these data suggest that the prophylactic antiviral effect of these IONPs may arise through their modulation of iron metabolism in the cells. By contrast, the cellular expression of TFRC, DMT1 and IREB2 was not significantly affected by treatment with Venofer, FeraSpin R and APS-IONP-10, and the expression of IREB2 was only mildly increased upon the treatment of cells with FAC (Fig. 9A).

Subsequently, Vero E6 cells were infected with SARS-CoV-2 over 24 h to determine whether the infection affects the expression of genes involved in iron metabolism and transport. Viral infection increased the expression of DMT1 three-fold (Fig. 9B), a divalent cation transporter involved in the transport of iron from the endosomes to the cytoplasm [60]. Viral infection also increased FPN1 expression by 38-fold (Fig. 9B), influencing the transport of iron out of the cell [43, 61], LCN2 expression seven-fold (Fig. 9B), which alters the iron availability and intracellular iron levels, and IREB2 expression by five-fold (Fig. 9B), affecting the expression of transcripts of genes involved in iron metabolism. In addition, viral infection decreased the expression of TFRC three-fold (Fig. 9B), the cell surface receptor necessary for cellular iron uptake through receptor-mediated endocytosis [62]. These data suggested that the transport of iron outside the cell is favored in SARS-CoV-2-infected cells and indeed, the concentration of intracellular iron measured by ICP-OES was reduced two-fold in SARS-CoV-2 infected cells relative to mock-infected cells (Fig. 9C), which could be a host response to inhibit viral infection.

It has been suggested the direct manipulation of iron metabolism by viruses and the consequences of imbalances in iron homeostasis caused by viruses is a critical aspect of COVID-19 pathogenesis [63]. As the outcome of ferroptosis is cell death, this might explain the clinical features of multiple organ involvement and failure in COVID-19 patients. Specifically, SARS-CoV-2-infected patients present high levels of ferritin and transferrin, diminishing the total amount of iron in the blood and provoking anemia [64, 65]. A preliminary study showed that the infection of Vero E6 cells with SARS-CoV-2 decreased their expression of GPX4, which encodes a protein that protects against iron-dependent ferroptotic cell death, suggesting an association between SARS-CoV-2 and ferroptosis [63]. Consequently, ferroptosis may occur due to the dysregulation of iron homeostasis in COVID-19 patients. Iron overload is an important mechanism that contributes to the pathogenesis of different viruses, such as hepatitis B, hepatitis C, HIV-1 and human cytomegalovirus infection [66–70]. Reducing the iron level in the infected cells can effectively inhibit the growth of these viruses and the development of the diseases induced by these viruses [71, 72].

To analyze whether the induction of FPN1, LCN2 and IREB2 expression caused by SARS-CoV-2 infection could somehow be counteracted by FAC, Venofer and IONP treatment, a mechanism that could underlie their antiviral effect, Vero E6 cells were infected with SARS-CoV-2 and 1 hpi, the cells were treated with the highest dose of FAC, Venofer or IONPs for a further 24 h. In most cases, treating infected cells with FAC, Venofer or IONPs changed the expression of the iron metabolism genes induced by SARS-CoV-2 infection to a profile that mainly resembled that of uninfected Vero E6 cells treated with these agents (Fig. 9D). Hence, the effect of FAC, Venofer or IONP treatment on iron metabolism might prevail over the effect of SARS-CoV-2 infection on these genes, probably reflected in the differences in the total intracellular iron found in cells treated with FAC (1.27 pg Fe/cell), Venofer (1.45 pg Fe/cell) or the different IONPs (FeraSpin R: 20.97 pg Fe/cell, APS-IONPs-10: 42.40 pg Fe/cell, DMSA-IONP-10: 41.53 pg Fe/cell and DMSA-IONP-16: 30.71 pg Fe/cell):(Additional file 1: Fig. S5)., and in untreated cells (0.19 pg Fe/cell: Fig. 9C). Moreover, SARS-CoV-2 infection did not significantly change the iron content observed in FAC, Venofer or IONP treated uninfected cells (Additional file 1: Fig. S5).

SARS-CoV-2 infection seems to favor the expression of FPN1 (38-fold increase) but the treatment of infected cells with FAC (four-fold), Venofer (three-fold), APS-IONP-10 (four-fold), and DMSA-IONP-16 (two-fold) significantly reduced FPN1 expression in such infected cells, whereas a 18- and ten-fold increase in the expression of FPN1 was maintained in SARS-CoV-2-infected cells after treatment with FeraSpin R and DMSA-IONP-10 (Fig. 9D). Thus, it seems that one consequence of the different treatments on SARS-CoV-2-infected cells is to counteract SARS-CoV-2-induced FPN1 expression. Furthermore, a similar situation was observed for IREB2 and DMT1, with stronger IREB2 expression in infected cells that remained untreated (five-fold) than in infected cells treated with FAC, Venofer or IONPs (less than two-fold for all the treatments). Similarly, the expression of DMT1 increased in the untreated, infected cells relative to the mock-infected cells (three-fold), whereas DMT1 expression did not increase in the infected cells treated with FAC, Venofer or IONPs (Fig. 9D). In the case of LCN2, although SARS-CoV-2 infection increased its expression seven-fold, infected cells treated with DMSA-coated IONPs enhanced the levels of LCN2 17- and ten-fold (Fig. 9D). In summary, the results from these experiments suggested that SARS-CoV-2 induced FPN1, DMT1, IREB2 and LCN2 expression, and that treatment with FAC, Venofer or IONPs can counteract the induction of FPN1, IREB2 and DMT1 expression, whereas the treatment of infected cells with DMSA-IONP-10 and DMSA-IONP-16 may enhance LCN2 expression.

Finally, the decreased levels of TFRC in infected cells treated with FAC, Venofer DMSA-IONP-10, DMSA-IONP-16 and FeraSpin R relative to untreated infected cells (Fig. 9D), the decreased levels of DMT1 in infected cells treated with FAC, Venofer and IONPs relative to untreated infected cells, and the increased levels of LCN2 in the infected cells treated with DMSA-IONP-10 and DMSA-IONP-16 relative to untreated infected cells, suggest that there may be less iron bioavailability at replication and transcription complexes in these cells, possibly underlying the antiviral effect of these IONPs.

## Conclusions

Having studied the potential of IONPs and IOHNPs for the treatment and/or prevention of SARS-CoV-2 infection, we conclude that IONPs and IOHNPs have a prophylactic and therapeutic effect against SARS-CoV-2 infection in Vero E6 cells at doses that are not cytotoxic. Moreover, both the therapeutic and prophylactic antiviral effects were dose-dependent. For the prophylactic effect, the highest non-cytotoxic concentrations of IONPs coated with DMSA were those that best inhibited viral replication. By contrast, for the therapeutic effect the highest concentrations of all the treatments reduced the viral titers at 24 and 48 hpi, being DMSA-IONP-10 and APS-IONP-10 those that best reduce the viral titers. Furthermore, treating the cells before or after the infection reduced viral replication and transcription in most cases. TEM analysis detected less intracellular and extracellular viral particles when the cells were treated with the DMSA-IONP-10 before or after infection, and DMVs, the sites for coronavirus replication and transcription, were hardly ever seen in the cells that were first treated with the IONPs and then infected. The decrease in viral titers, as well as the decrease in viral replication and transcription, suggests that IONPs and IOHNPs could interfere with viral load and therefore, viral pathogenesis.

FeraSpin R, DMSA-IONP-10 and DMSA-IONP-16 can induce significant oxidative stress in Vero E6 cells, activating different antioxidant effects, with DMSA-IONP-10 and DMSA-IONP-16 the NPs that induce the highest levels of oxidative stress. The weaker antiviral activity in the presence of NAC, a ROS scavenger, suggested that the induction of oxidative stress in the cells by these DMSA-IONPs was at least partially responsible for the antiviral effect they produced. IONPs and IOHNPs also affect the expression of genes involved in iron metabolism and transport in Vero E6 cells. SARS-CoV-2 infection enhances the expression genes involved in iron metabolism and transport, such as FPN1, DMT1, LCN2 and IREB2. Treatment of cells with FAC, Venofer or IONPs can counteract the induction of FPN1, DMT1 and IREB2 expression to a greater or lesser extent in the infected cells.

In summary, we showed that the treatment of cells with IONPs and IOHNPs decreases SARS-CoV-2 production, affects the regulation of cellular iron metabolism in the cells, and that treatment of SARS-CoV-2 infected cells with FAC, Venofer or IONPs could counteract the transcription of FPN1, DMT1 and IREB2 induced by the virus, which could be a factor that prevents the correct replication of the virus after infection of Vero E6 cells. In addition, another factor that could contribute synergistically to the antiviral effect of IONPs is the induction of oxidative stress. Our data suggest that the induction of oxidative stress after the treatment of cells with the IONPs is due firstly to the coating (DMSA) and secondarily, to the fact that oxidative stress is triggered as a consequence of the degradation of the iron core. Together these results suggest that the IONPs and IOHNPs, may be repurposed and used as prophylactic and therapeutic treatments against SARS-CoV-2 infection.

## Supplementary Information

**Additional file 1: Figure S1.** Physicochemical characterization of DMSA-IONP-5. **Figure S2.** Ultrastructural analysis of infected cells treated with different IONPs. **Figure S3.** Viability and internalization of IONPs in Vero E6 cells treated with N-acetylcysteine. **Figure S4.** ROS generation observed through DHR fluorescence in the presence and absence of the antioxidant N-acetylcysteine. **Figure S5.** The concentrations of intracellular iron in mock-infected and SARS-CoV-2 infected cells treated with FAC, Venofer or IONPs (100 or 250 µg Fe/ml), as measured by ICP-OES.

**Additional file 2: **The excel file (.xls) shows all the data obtained from this research.

## Data Availability

All data generated or analyzed during this study are included in this published article and its Additional files (Additional file 1 and Additional file 2).
