# Supplementary material for: Iron oxide and iron oxyhydroxide nanoparticles impair SARS-CoV-2 infection of cultured cells
Source: J Nanobiotechnology. 2022 Jul 30;20:352. doi: 10.1186/s12951-022-01542-2 (PMC9338509; doi:10.1186/s12951-022-01542-2)
Supplement: Supplementary file 1 — Additional file 1: Figure S1. Physicochemical characterization of DMSA-IONP-5. Figure S2. Ultrastructural analysis of infected cells treated with different IONPs. Figure S3. Viability and internalization of IONPs in Vero E6 cells treated with N-acetylcysteine. Figure S4. ROS generation observed through DHR fluorescence in the presence and absence of the antioxidant N-acetylcysteine. Figure S5. The concentrations of intracellular iron in mock-infected and SARS-CoV-2 infected cells treated with FAC, Venofer or IONPs (100 or 250 µg Fe/ml), as measured by ICP-OES. [file 12951_2022_1542_MOESM1_ESM.pdf]

Additional file 1

Iron oxide and iron oxyhydroxide nanoparticles impair SARS-CoV-2 infection of cultured cells

Marta L. DeDiego[1#*], Yadileiny Portilla[2#], Neus Daviu[2], Darío López-García[1], Laura Villamayor[1†], Vladimir Mulens-Arias[2§], Jesús G. Ovejero[3], Álvaro Gallo-Cordova[3], Sabino Veintemillas-Verdaguer[3], M. Puerto Morales[3*] and Domingo F. Barber[2*]

[1]*Department of Molecular and Cellular Biology, Centro Nacional de Biotecnología (CNB-CSIC), Darwin 3, 28049 Madrid, Spain.*

[2]*Department of Immunology, Oncology and Nanobiomedicine Initiative, Centro Nacional de Biotecnología (CNB-CSIC), Darwin 3, 28049 Madrid, Spain.*

[3]*Department of Energy, Environment and Health, Instituto de Ciencia de Materiales de Madrid (ICMM-CSIC), Sor Juana Inés de la Cruz 3, 28049 Madrid, Spain.*

[†]*Current address: Instituto de Investigaciones Biomédicas "Alberto Sols" (IIBm-CSIC-UAM), Arturo Duperier 4, 28029 Madrid, Spain.*

[§]*Current address: Integrative Biomedical Materials and Nanomedicine Lab, Department of Experimental and Health Sciences (DCEXS), Pompeu Fabra University, PRBB, Carrer Doctor Aiguader 88, 08003 Barcelona, Spain.*

Author Contributions: [#]*M.L.D. and Y.P. contributed equally to this work.*

Corresponding and Senior Authors: **E-mail:* dfbarber@cnb.csic.es *(D.F.B.);* puerto@icmm.csic.es *(M.P.M.);* marta.lopez@cnb.csic.es *(M.L.D.)*

Description and characterization of DMSA-IONP-5

A reference sample for comparison has been used based on DMSA coated iron oxide nanoparticles (DMSA-IONPs) that are 5 ± 1.1 nm in diameter, prepared by thermal decomposition in organic media (**Fig. S1A and S1B**). Thermogravimetric (TG) analysis of the IONP samples shows the different organic content as a function of the particle size. An increase in the amount of DMSA from 9% up to 20% and 36% has been measured for particles of 5, 10 and 16 nm, respectively (**Fig. S1C**). The iron leaching was determined for the DMSA-IONPs in different conditions of pH and temperature. In all cases the dissolution rate is very low (< 5%) when compared to Venofer (~ 38 %), yet as the particle size decreases the dissolution rate increases clearly from < 0.5% to 2.8% (**Fig. S1D**).

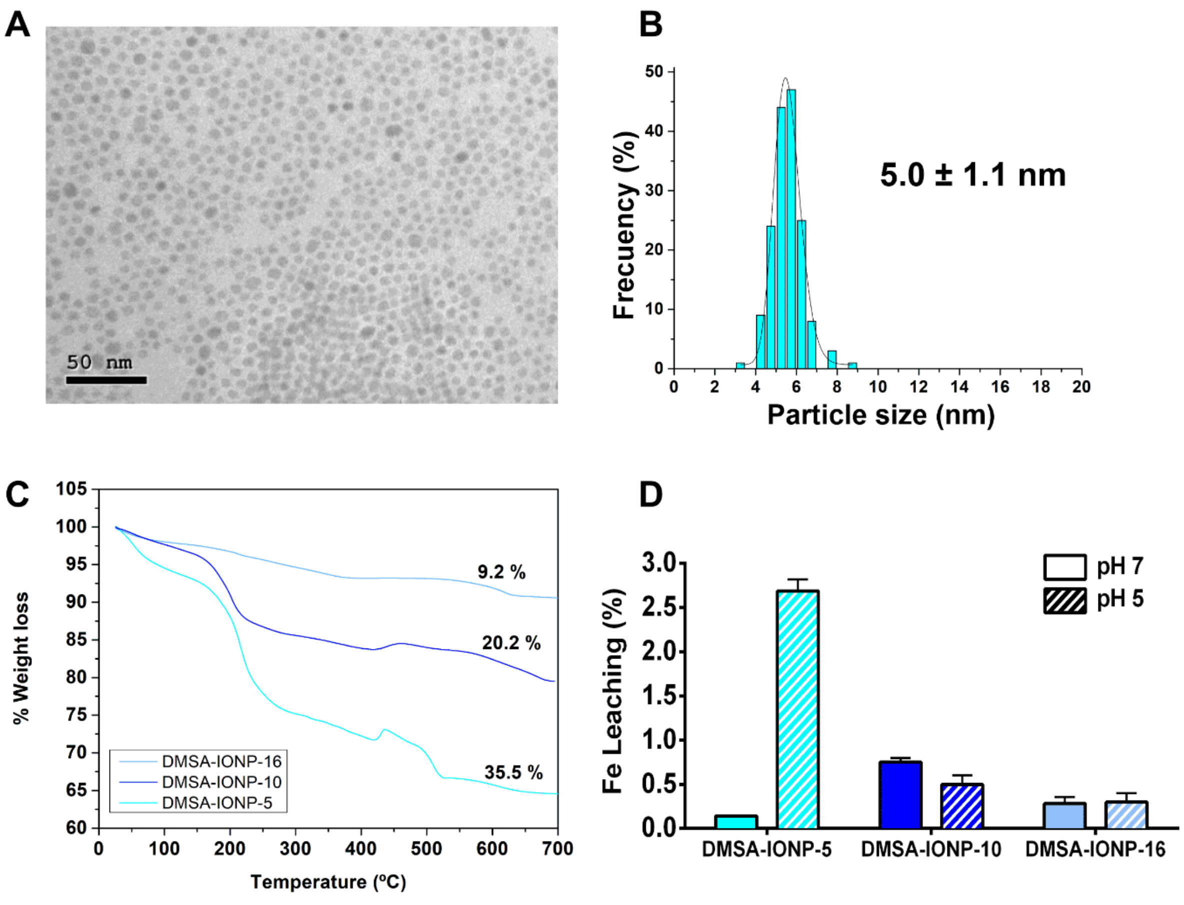

Fig. S1. Physicochemical characterization of DMSA-IONP-5. (A) TEM image of DMSA-IONP-5, scale bar 50 nm. (B) The distribution and Gaussian fitting of the nanoparticle size. (C) Thermogravimetric analysis (TG) of three DMSA-IONPs. (D) Comparative iron leaching from the DMSA-IONPs under the specified conditions (pH 7 or 5) depending on the size.

Structural changes to SARS-CoV-2-infected cells

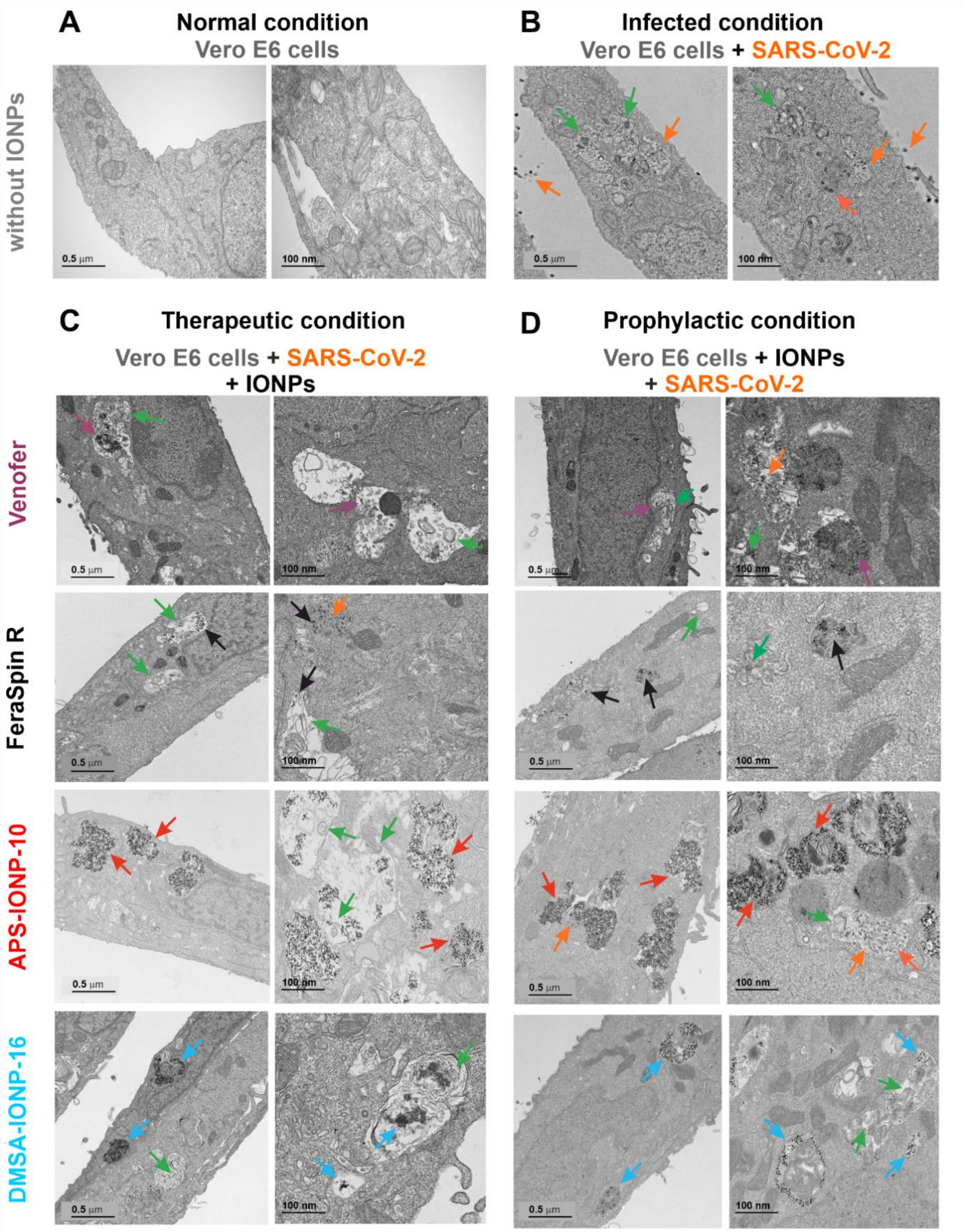

Fig. S2. Ultrastructural analysis of infected cells treated with different IONPs. (A) Normal condition: untreated and mock-infected Vero E6 cells (Control). (B) Infected condition: Vero E6 cells were infected at a MOI of 0.001 with SARS-CoV-2 for 24 h. (C) The cells were infected with SARS-CoV-2 and 1 hpi the cells were treated with IONPs at 100 (Venofer) or 250 µg Fe/ml (FeraSpin R, APS-IONP-10 and DMSA-IONP-16) or alternatively, (D) the cells were treated with IONPs and then infected. In all cases the cells were processed for TEM of ultrathin sections at 24 hpi. Colored arrows indicate the presence of viral particles (in orange), DMVs (in green) and the accumulation of IONPs inside the Vero E6 cells: Venofer (in purple), FeraSpin R (in black), APS-IONP-10 (in red) and DMSA-IONP-16 (light blue). Scale bars: 0.5 µm and 100 nm, as indicated.

Cell viability studies to determine the optimal concentration of the anti-oxidant N-acetylcysteine

To select the optimal non-toxic dose of N-acetylcysteine (NAC) we performed a PrestoBlue assay treating Vero E6 cells with concentrations between 0-200 µM of NAC. Cell viability in the PrestoBlue assay was more than 90% between 125 µM (98.28%) and 250 µM (93.53%), and therefore, we chose 200 µM as the optimal working dose for NAC (**Fig. S3A**). In addition, it was verified by an ICP-OES assay that NAC treatment did not significantly affect the internalization of FAC, Venofer, and the IONPs (**Fig. S3B)**.

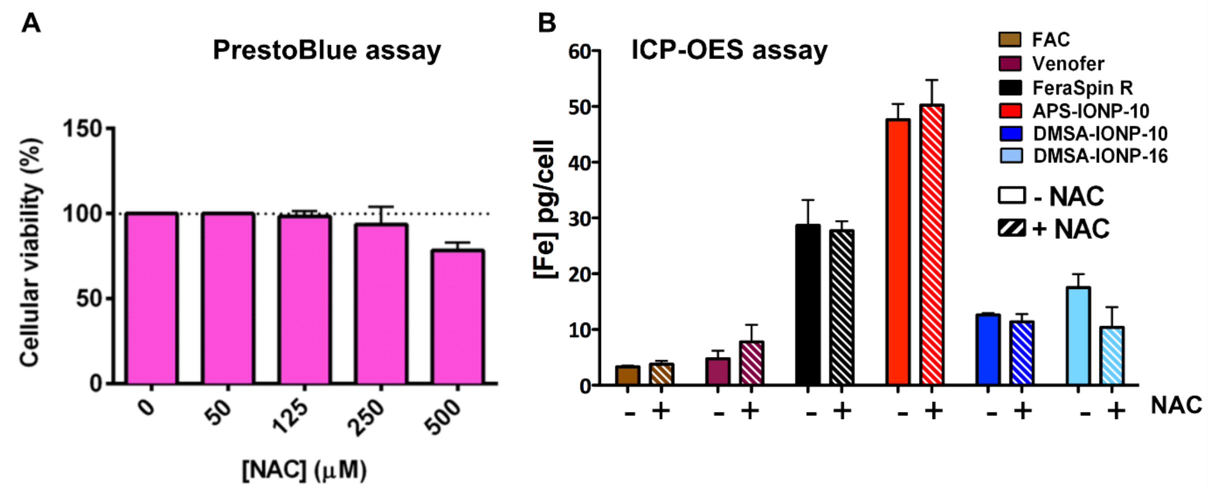

Fig. S3. Viability and internalization of IONPs in Vero E6 cells treated with N-acetylcysteine. (A) Concentration dependent cytotoxic effects of a 24 h incubation with NAC as measured with the PrestoBlue assay. (B) Evaluation of FAC, Venofer, FeraSpin R, APS-IONP-10, DMSA-IONP-10 and DMSA-IONP-16 internalization by Vero E6 cells after treatment with NAC (at 200 µM) and IONPs (at the highest doses). The data (mean ± SD) are representative of three independent experiments.

Verification of the anti-oxidant activity of N-acetylcysteine

To verify that NAC inhibits the production of reactive oxygen species (ROS), we performed a dihydrorhodamine 123 (DHR) assay in which we quantified the amount of ROS produced in the presence and absence of NAC (**Fig. S4A**). The results showed that NAC reverses the production of ROS in response to FAC and all the IONPs with exception of APS-IONP-10,

observing a decrease in the ROS produced by Vero E6 cells previously treated with the antioxidant NAC (**Fig. S4B**).

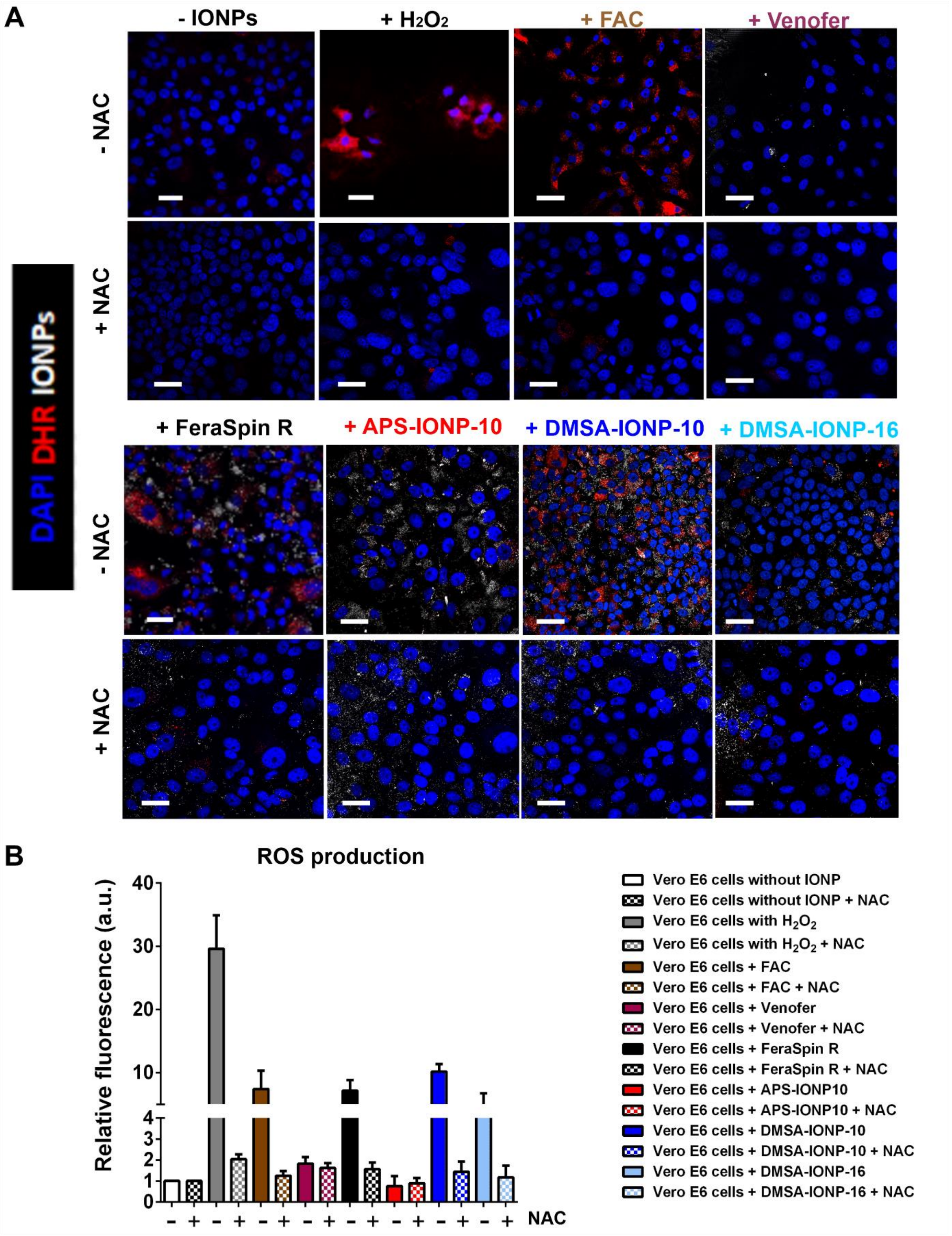

Fig. S4. ROS generation observed through DHR fluorescence in the presence and absence of the antioxidant N-acetylcysteine. (A) ROS production in Vero E6 cells treated with FAC, Venofer and DMSA-IONPs, with and without NAC, as observed through the DHR assay. (B) Quantitative image

analysis of DHR fluorescence intensity using Image J software. The data are the mean ± SD from two independent experiments.

Intracellular iron in mock-infected and SARS-CoV-2 infected cells after treatment with FAC, Venofer or IONPs

To check whether the amount of intracellular iron varied after treatment with the high dose of FAC, Venofer or IONPs in cells infected by SARS-CoV-2, the amount of iron was quantified using ICP-OES and expressed relative to the uninfected cells. These results showed that whereas after treatment with FAC and Venofer, the amount of intracellular iron decreased in infected cells (0.5-fold and 0.7-fold, respectively), the iron concentration in infected cells after IONP treatment compared to non-infected Vero E6 cells was similar (**Fig. S5**).

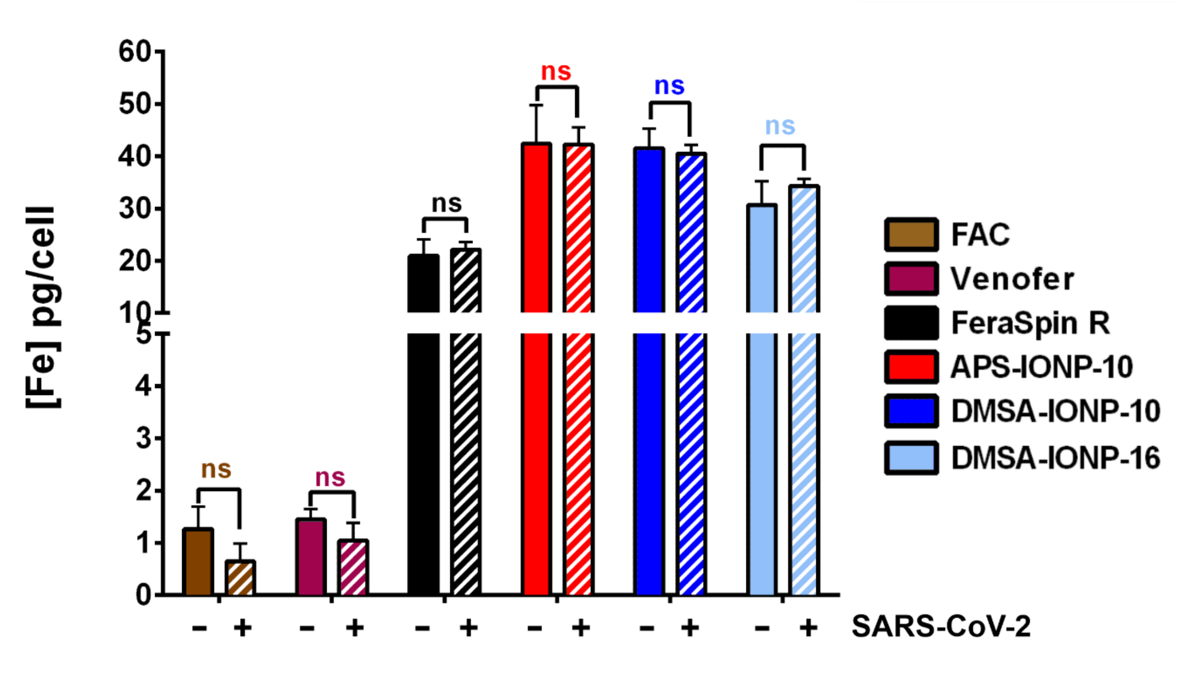

Fig. S5. The concentrations of intracellular iron in mock-infected and SARS-CoV-2 infected cells treated with FAC, Venofer or IONPs (100 or 250 µg Fe/ml), as measured by ICP-OES. The data are the mean ± SD of three independent experiments. Two-way ANOVA followed by Tukey multiple comparison test: *p < 0.05; **p < 0.01; ***p < 0.001; and ns, not significant.
